# Supplementary material for: The prevalence of mental disorders among homeless people in high-income countries: An updated systematic review and meta-regression analysis
Source: PLoS Med. 2021 Aug 23;18(8):e1003750. doi: 10.1371/journal.pmed.1003750 (PMC8423293; doi:10.1371/journal.pmed.1003750)
Supplement: S1 Table — (DOCX) [file pmed.1003750.s001.docx]

| S1 Table. Data Base Search Strings | | |
| --- | --- | --- |
| Search Engine – Data Base | **Search Query** | **Results** |
| Google Scholar | *(Homeless OR shelter) („mental disease“ OR „mental disorder“ OR „mentally ill“ OR „mental illness“ OR alcohol OR drug OR psychotic OR schizophrenia OR depression OR „personality disorder“) (prevalence OR morbidity)* | 1000 |
|  | First 1000 Results  Custom Range 2008-2021  Assessed on 01/04/21, 11:00 |  |
| Google Scholar | *The prevalence of mental disorders among the homeless in western countries: systematic review and meta-regression analysis – search within citing articles* | 959 |
|  | Assessed on 01/04/21, 11:30 |  |
| OvidSP – Embase | *1. mental disease.mp. or exp mental disease/*  *2. mental disorder*.mp.*  *3. psychiatric disorder*.mp.*  *4. mental ilness*.mp.*  *5. mentally ill.mp.*  *6. mental ill - heath.mp.*  *7. mental health.mp. or exp mental health/*  *8. exp depression/ or depressi*.mp.*  *9. Affective disorder*.mp.*  *10. exp mood disorder/ or mood disorder*.mp.*  *11. bipolar.mp. or exp bipolar disorder/ or exp bipolar depression/ or exp bipolar mania/ or exp bipolar II disorder/*  *12. exp schizophrenia/ or schizophren*.mp.*  *13. Psychotic*.mp.*  *14. psychosis*.mp. or exp psychosis/ or exp acute psychosis/ or exp schizoaffective psychosis/ or exp puerperal psychosis/*  *15. exp "substance use"/ or "substance use*".mp.*  *16. substance abuse.mp. or exp substance abuse/*  *17. exp drug dependence/ or drug dependen*.mp.*  *18. exp "drug use"/ or "drug use*".mp.*  *19. drug abuse.mp. or exp drug abuse/*  *20. exp alcohol withdrawal seizure/ or exp alcohol/ or exp alcohol withdrawal syndrome/ or alcohol.mp. or exp alcohol abuse/ or exp alcohol psychosis/*  *21. exp personality disorder/ or personality disorder*.mp.*  *22. exp homeless youth/ or exp homeless person/ or homeless*.mp.*  *23. roofless*.mp.*  *24. exp homelessness/*  *25. shelter*.mp.*  *26. living on the street*.mp.*  *27. substance-related disorder*.mp.*  *28. alcoholism.mp. or exp alcoholism/*  *29. 22 or 23 or 24 or 25 or 26*  *30. 1 or 2 or 3 or 4 or 5 or 6 or 7 or 8 or 9 or 10 or 11 or 12 or 13 or 14 or 15 or 16 or 17 or 18 or 19 or 20 or 21 or 27 or 28*  *31. prevalence.mp. or exp prevalence/*  *32. morbidity.mp. or exp morbidity/*  *33. exp epidemiology/ or epidemiology.mp.*  *34. 31 or 32 or 33*  *35. 29 and 30 and 34*  *36. limit 35 to yr="2008-current"* | 3664 |
|  | Assessed on 01/04/21, 12:00 |  |
| OvidSP – MEDLINE | *1. exp Mental Disorders/ or mental disorder*.mp.*  *2. mental disease*.mp.*  *3. psychiatric disorder*.mp.*  *4. mental illness*.mp.*  *5. mentally ill.mp. or exp Mentally Ill Persons/*  *6. mental health.mp. or exp Mental Health/*  *7. exp Depressive Disorder/ or exp Depressive Disorder, Major/ or exp Depression/ or depressi*.mp.*  *8. affective disorder*.mp.*  *9. exp Mood Disorders/ or mood disorder*.mp.*  *10. exp BIPOLAR DISORDER/ or bipolar.mp.*  *11. exp Schizophrenia/ or schizophren*.mp.*  *12. exp PSYCHOTIC DISORDERS/ or psychotic*.mp.*  *13. psychosis*.mp.*  *14. exp Substance-Related Disorders/ or "substance use*".mp.*  *15. substance abuse*.mp.*  *16. "drug use*".mp.*  *17. drug dependen*.mp.*  *18. substance related disorder*.mp.*  *19. exp ALCOHOL-RELATED DISORDERS/ or alcohol*.mp.*  *20. exp Psychotic Disorders/ or exp Borderline Personality Disorder/ or exp Personality Disorders/ or personality disorder*.mp.*  *21. exp Homeless Youth/ or exp HOMELESS PERSONS/ or homeless*.mp.*  *22. homelessness*.mp.*  *23. roofless*.mp.*  *24. shelter*.mp.*  *25. living on the street*.mp.*  *26. alcoholism.mp. or exp ALCOHOLISM/*  *27. 21 or 22 or 23 or 24 or 25*  *28. 1 or 2 or 3 or 4 or 5 or 6 or 7 or 8 or 9 or 10 or 11 or 12 or 13 or 14 or 15 or 16 or 17 or 18 or 19 or 20 or 26*  *29. exp PREVALENCE/ or prevalence.mp.*  *30. morbidity.mp. or exp MORBIDITY/*  *31. exp EPIDEMIOLOGY/ or epidemiology.mp.*  *32. 29 or 30 or 31*  *33. 27 and 28 and 32*  *34. limit 33 to yr="2008-current"* | 2358 |
|  | Assessed on 01/04/21, 12:00 |  |
| PubMed – MEDLINE | *(Mental disorder* OR „Mental Disorders“[MeSH] OR Mental disease* OR Psychiatric disorder* OR Mental illness* OR Mentally ill OR Mental health OR „Mental Health“[MeSH] OR Depressi* OR „Depression“[MeSH] OR „Depressive Disorder“[MeSH] OR Affective disorder* OR Mood disorder* OR „Mood Disorders“[MeSH] OR Bipolar OR „Bipolar Disorder“[MeSH] OR Schizophren* OR „Schizophrenia“[MeSH] OR „Schizophrenia Spectrum and Other Psychotic Disorders“[MeSH] OR Psychotic* OR Psychosis* OR „Psychotic Disorders“[MeSH] OR „Substance use“ OR „Substance-Related Disorders“[MeSH] OR Substance abuse OR „Drug use“ OR Drug abuse OR Drug dependen* OR Substance-related disorder* OR Alcoholism OR „Alcohol-Related Disorders“[MeSH] OR „Alcoholism“[MeSH] OR Personality disorder* OR „Personality Disorders“) AND (homeless* OR „Homeless Persons“[MeSH] OR roofless* OR shelter* OR living on the street*) AND ("Morbidity"[Mesh] OR morbidit* OR "Prevalence"[Mesh] OR prevalenc* OR epidemiolog* OR "Epidemiology"[Mesh])*  *From 2008/01/01 to 2021/04/01* | 3644 |
|  | Assessed on 01/04/21, 12:30 |  |
| EBSCOhost – PsycINFO | *((DE "Mental Disorders") OR mental disorder* OR mental disease* OR psychiatric disorder* OR mental illness* OR mentally ill OR (DE "Mental Health") OR mental health OR depress* OR DE ("Depression (Emotion)") OR Affective disorder* OR Mood disorder* OR bipolar OR DE ("Bipolar Disorder") OR schizophren* OR (DE "Schizophrenia") OR psychotic* OR psychosis* OR (DE "Psychosis") OR (DE "Substance Use Disorder") OR Substance use* OR Substance abuse OR Drug use* OR Drug abuse OR Drug dependen* OR Substance related disorder OR Alcoholism OR (DE "Alcoholism") OR Alcohol* OR Personality Disorder* OR (DE "Personality Disorders")) AND (homeless* OR (DE “Homeless”) OR roofless* OR shelter* OR (DE “Shelters”) OR living on the street*) AND (morbidit* OR prevalenc* OR (DE "Epidemiology") OR (DE "Morbidity"))*  *From 2008/01/01 to 2021/04/01* | 776 |
|  | Assessed on 01/04/21, 12:30 |  |
