# Supplementary material for: The prevalence of mental disorders among homeless people in high-income countries: An updated systematic review and meta-regression analysis
Source: PLoS Med. 2021 Aug 23;18(8):e1003750. doi: 10.1371/journal.pmed.1003750 (PMC8423293; doi:10.1371/journal.pmed.1003750)
Supplement: S2 Table — (DOCX) [file pmed.1003750.s002.docx]

| **Section/topic** | **#** | **Checklist item** | **Reported on page #** |
| --- | --- | --- | --- |
| **TITLE** | | |  |
| Title | 1 | Identify the report as a systematic review, meta-analysis, or both. | Title:  “The prevalence of mental disorders among homeless people in high-income countries: updated systematic review and meta-regression analysis” |
| **ABSTRACT** | | |  |
| Structured summary | 2 | Provide a structured summary including, as applicable: background; objectives; data sources; study eligibility criteria, participants, and interventions; study appraisal and synthesis methods; results; limitations; conclusions and implications of key findings; systematic review registration number. | Abstract:  “Background: Homelessness continues to be a pressing public health concern in many countries, and mental disorders in homeless persons contribute to their high rates of morbidity and mortality. Many primary studies have estimated prevalence rates for mental disorders in homeless individuals. We conducted a systematic review and meta-analysis of studies on the prevalence of any mental disorder, and major psychiatric diagnoses in clearly defined homeless populations in any high-income country (…)” |
| **INTRODUCTION** | | |  |
| Rationale | 3 | Describe the rationale for the review in the context of what is already known. | Introduction, paragraph 3:  “Information on the overall extent and pattern of mental disorders among homeless people are necessary to inform resource allocation and service development, and allow for consideration of evidence gaps with researchers, clinicians, and policymakers. The large number of primary studies, of varying quality and samples, means that systematic reviews are required to clarify and synthesize the evidence, underscore main findings, and consider implications (…)” |
| Objectives | 4 | Provide an explicit statement of questions being addressed with reference to participants, interventions, comparisons, outcomes, and study design (PICOS). | Introduction, paragraph 3:  “Thus, we conducted an updated meta-analysis on the prevalence of mental disorders among homeless people in high-income countries (…)” |
| **METHODS** | | |  |
| Protocol and registration | 5 | Indicate if a review protocol exists, if and where it can be accessed (e.g., Web address), and, if available, provide registration information including registration number. | Methods, paragraph 1:  “The protocol for this systematic review and meta-analysis has been published (PROSPERO registration no. CRD42018085216)” |
| Eligibility criteria | 6 | Specify study characteristics (e.g., PICOS, length of follow-up) and report characteristics (e.g., years considered, language, publication status) used as criteria for eligibility, giving rationale. | Methods, paragraphs 2-3:  “Inclusion criteria were: (…)”  “Surveys with (…) were excluded.” |
| Information sources | 7 | Describe all information sources (e.g., databases with dates of coverage, contact with study authors to identify additional studies) in the search and date last searched. | Methods, paragraph 1:  “We have updated an earlier review (27) which was based on a search for articles published up until December 2007, so we targeted new primary studies published between January 2008 and March 2021. We searched Embase via OvidSP, MEDLINE via OvidSP and via PubMed, and PsycINFO via EBSCOhost. Additionally, we used Google Scholar using a search term and screened all literature citing the previous review. Finally, we screened reference lists of relevant publications.” |
| Search | 8 | Present full electronic search strategy for at least one database, including any limits used, such that it could be repeated. | Methods, paragraph 1:  “Each search employed a specific combination of search terms designed to fit the databases’ respective syntaxes and thesaurus systems (S1 Table).” |
| Study selection | 9 | State the process for selecting studies (i.e., screening, eligibility, included in systematic review, and, if applicable, included in the meta-analysis). | Methods, paragraph 4:  “In order to assess all results from the bibliographic search process, researchers SS, SG and KD each carried out a multi-level screening process independently from one another. Any differences between results were resolved by consensus between all the authors.“ |
| Data collection process | 10 | Describe method of data extraction from reports (e.g., piloted forms, independently, in duplicate) and any processes for obtaining and confirming data from investigators. | Methods, paragraph 5:  “If data regarding any of these categories was unclear in the published study, we corresponded with primary study authors.”  Methods, paragraph 6:  “This process was carried out by SS, SG and KD independently and any differences were resolved by discussion.” |
| Data items | 11 | List and define all variables for which data were sought (e.g., PICOS, funding sources) and any assumptions and simplifications made. | Methods, paragraph 5:  “Information from included surveys was extracted on (…)” |
| Risk of bias in individual studies | 12 | Describe methods used for assessing risk of bias of individual studies (including specification of whether this was done at the study or outcome level), and how this information is to be used in any data synthesis. | Methods, paragraph 6:  “Each included publication was rated on methodological quality by two sets of criteria specifically designed to assess prevalence studies: the Joanna Briggs Institute Critical Appraisal Tool for Prevalence Studies (34) and a Risk of Bias Tool (35)” |
| Summary measures | 13 | State the principal summary measures (e.g., risk ratio, difference in means). | Methods, paragraph 7:  “Prevalence estimates were transformed on the double arcsine function in order to avoid variance instability and confidence intervals exceeding the interval (0 ≤ x ≤ 1), in which prevalence proportions can be meaningfully defined (…)” |
| Synthesis of results | 14 | Describe the methods of handling data and combining results of studies, if done, including measures of consistency (e.g., I^2^) for each meta-analysis. | Methods, paragraph 7:  “ We calculated random effects models, which we deemed appropriate considering sampling differences”  “To quantify measures of between-study-heterogeneity, we report the test statistic QE and corresponding p-value as well as the I2 statistic. Additionally, we calculated 95% prediction intervals for all meta-analytical models (42)” |

| **Section/topic** | **#** | **Checklist item** | **Reported on page #** |
| --- | --- | --- | --- |
| Risk of bias across studies | 15 | Specify any assessment of risk of bias that may affect the cumulative evidence (e.g., publication bias, selective reporting within studies). | Methods, paragraph 8:  “Additional meta-analyses were carried out in each diagnostic category for Low Risk of Bias studies, assigned during quality assessment (35). Subgroup analyses comparing Low Risk of Bias and Moderate Risk of Bias studies were performed through a Q-test. In cases of significant between-subgroup difference, a meta-regression model with Risk of Bias assessment as a single independent variable was computed to estimate the proportion of variance explained by disparities in methodological quality. “ |
| Additional analyses | 16 | Describe methods of additional analyses (e.g., sensitivity or subgroup analyses, meta-regression), if done, indicating which were pre-specified. | Methods, paragraph 8:  “For each diagnostic category, meta-regression analyses were performed to investigate potential sources of heterogeneity. Continuous independent variables for single factor meta-regression were (…)” |
| **RESULTS** | | |  |
| Study selection | 17 | Give numbers of studies screened, assessed for eligibility, and included in the review, with reasons for exclusions at each stage, ideally with a flow diagram. | Results, paragraph 1:  “We identified a total of 39 studies comprising data on 8049 homeless individuals (…)” |
| Study characteristics | 18 | For each study, present characteristics for which data were extracted (e.g., study size, PICOS, follow-up period) and provide the citations. | Results, paragraph 2:  “S4 Table provides further information on methodological and sample characteristics” |
| Risk of bias within studies | 19 | Present data on risk of bias of each study and, if available, any outcome level assessment (see item 12). | Results, paragraph 2:  “For quality ratings, see S5 Table and S6 Table.” |
| Results of individual studies | 20 | For all outcomes considered (benefits or harms), present, for each study: (a) simple summary data for each intervention group (b) effect estimates and confidence intervals, ideally with a forest plot. | Results, paragraphs 3-10  See figures 2-8 |
| Synthesis of results | 21 | Present results of each meta-analysis done, including confidence intervals and measures of consistency. | Results, paragraphs 3-10:  “Homeless people having at least one diagnosis of a current mental disorder were based on 8 surveys (28,51,54,62,71–73,81) with a random effects pooled prevalence estimated at 76.2% (95% CI 64.0% - 86.6%) (Fig 2).”  *(and equivalent statements for all following diagnostic categories)* |
| Risk of bias across studies | 22 | Present results of any assessment of risk of bias across studies (see Item 15). | Results, paragraphs 3-10:  “In a subgroup-analysis of four Low Risk of Bias studies (62,71,73,81), the random effects prevalence was 75.3% (95% CI 50.2% - 93.6%; I2= 81% [95% CI 32% - 99%]). There was no significant difference between quality subgroups (Q=0.03, p=0.87).”  *(and equivalent statements for all following diagnostic categories)* |
| Additional analysis | 23 | Give results of additional analyses, if done (e.g., sensitivity or subgroup analyses, meta-regression [see Item 16]). | Pages 8-12:  “Univariable meta-regression analysis revealed that studies with randomized sampling procedures reported significantly higher prevalence estimates than ones with other sampling procedures, accounting for a large proportion of heterogeneity (R2=59%) (see S8 Table). Sampling procedure was chosen as the only predictor variable by multivariable model selection (see Table 1).”  *(and equivalent statements for all following diagnostic categories)* |
| **DISCUSSION** | | |  |
| Summary of evidence | 24 | Summarize the main findings including the strength of evidence for each main outcome; consider their relevance to key groups (e.g., healthcare providers, users, and policy makers). | Discussion, paragraphs 2-4:  “With a pooled prevalence of around 35%, alcohol-related disorders were the most prevalent diagnosis (…)”  “A second main finding was that some study characteristics consistently explained the variations in prevalence (…)”  “Thirdly, we found high prevalence rates for treatable mental illnesses, with one in eight with either major depression (12.6%) or schizophrenia spectrum disorders (12.4%). (…)” |
| Limitations | 25 | Discuss limitations at study and outcome level (e.g., risk of bias), and at review-level (e.g., incomplete retrieval of identified research, reporting bias). | Discussion, paragraphs 7-8:  “Some limitations to this review need to be considered (…)” |
| Conclusions | 26 | Provide a general interpretation of the results in the context of other evidence, and implications for future research. | Discussion, paragraph 9:  “Future research should focus on integrated service models addressing the identified needs of substance use disorders, schizophrenia spectrum disorders, and depression in the homeless as a priority. In addition, new work could consider focusing on underrepresented subpopulations like homeless women and migrants. Furthermore, longitudinal studies could examine mechanisms linking homelessness and mental disorders in order to develop more effective preventive measures.” |
| **FUNDING** | | |  |
| Funding | 27 | Describe sources of funding for the systematic review and other support (e.g., supply of data); role of funders for the systematic review. | See “Financial Disclosure” Section |
