# Supplementary material for: The prevalence of mental disorders among homeless people in high-income countries: An updated systematic review and meta-regression analysis
Source: PLoS Med. 2021 Aug 23;18(8):e1003750. doi: 10.1371/journal.pmed.1003750 (PMC8423293; doi:10.1371/journal.pmed.1003750)
Supplement: S3 Table — (DOCX) [file pmed.1003750.s003.docx]

| **S3 Table. Studies excluded at full-text level screening with reasons** | | |
| --- | --- | --- |
| ***#*** | ***Study*** | ***Eligibility Criteria not met*** |
| **1** | Argintaru N, Chambers C, Gogosis E, Farrell S, Palepu A, Klodawsky F, et al. A cross-sectional observational study of unmet health needs among homeless and vulnerably housed adults in three Canadian cities. BMC Public Health. 2013;13(13):577. | Incl. 2) No diagnoses by ICD or DSM criteria |
| **2** | Aubry T, Duhoux A, Klodawsky F, Ecker J, Hay E. A longitudinal study of predictors of housing stability, housing quality, and mental health functioning among single homeless individuals staying in emergency shelters. Am J Community Psychol. 2016 Sep;58(1–2):123–35. | Incl. 2) No diagnoses by ICD or DSM criteria |
| **3** | Bacciardi S, Maremmani G, Nikoo N, Cambioli L, Schutz C, Jang K. Is bipolar disorder associated with tramautic brain injury in the homeless? Riv Psichiatr. 2017;52(1):40–6. | Excl. 2) Selected subpopulation – only homeless individuals with a diagnosis of mental disorder |
| **4** | Barrett P, Griffin E, Corcoran P, O’Mahony MT. Self-harm among the homeless population in Ireland: A national registry-based study of incidence and associated factors. J Affect Disord. 2018;229:523–31. | Incl. 2) No diagnoses by ICD or DSM criteria  Incl. 4) Registry study format does not provide point prevalence or 12-months-prevalence rates  Excl. 2) Selected subpopulation – only homeless individuals who presented to emergency departments |
| **5** | Beijer U, Andréasson S. Gender, hospitalization and mental disorders among homeless people compared with the general population in Stockholm. Eur J Public Health. 2010 Oct;20(5):511–6. | Incl. 4) Registry study format does not provide point prevalence or 12-months-prevalence rates  Excl. 2) Selected subpopulation – only homeless individuals treated in public healthcare facilities |
| **6** | Braun E, Gazdag G. [Prevalence of psychiatric disorders in homeless population]. Psychiatr Hung. 2015;30(1):60–7. | Excl. 1) Response rate at 36% |
| **7** | Burns A, Robins A, Hodge M, Holmes A. Long-term homelessness in men with a psychosis: Limitation of services. Int J Ment Health Nurs. 2009 Apr;18(2):126–32. | Incl. 2) No diagnoses by ICD or DSM criteria  Excl. 2) Selected subpopulation – only homeless individuals with a diagnosis of mental disorder |
| **8** | Calvo F, Fitzpatrick S, Fabregas C, Carbonell X, Group C, Turro-Garriga O. Individuals experiencing chronic homelessness: A 10-year follow-up of a cohort in Spain. Health Soc Care Community. 2020;28(5):1787–94. | Incl. 2) No diagnoses by ICD or DSM criteria  Incl. 4) Longitudinal design does not provide point prevalence or 12-months-prevalence rates |
| **9** | Chambers C, Chiu S, Scott AN, Tolomiczenko G, Redelmeier DA, Levinson W, et al. Factors associated with poor mental health status among homeless women with and without dependent children. Community Ment Health J. 2014 Jul;50(5):553–9. | Incl. 2) No diagnoses by ICD or DSM criteria |
| **10** | Cherner RA, Farrell S, Hwang SW, Aubry T, Klodawsky F, Hubley AM, et al. An investigation of predictors of mental health in single men and women experiencing homelessness in three Canadian cities. J Soc Distress Homeless. 2018;27(1):25–33. | Incl. 2) No diagnoses by ICD or DSM criteria  Incl. 3) Assessment by self-report measure |
| **11** | Chong MT, Yamaki J, Harwood M, D’Assalenaux R, Rosenberg E, Aruoma O, et al. Assessing health conditions and medication use among the homeless community in Long Beach, California. J Res Pharm Pract. 2014;3(2):56–61. | Incl. 2) No diagnoses by ICD or DSM criteria  Incl. 3) Assessment by self-report measure  Incl. 4) Point prevalence or 12-month prevalence rates not provided |
| **12** | Churchard A, Ryder M, Greenhill A, Mandy W. The prevalence of autistic traits in a homeless population. Autism. 2018;23(3):665–76. | Incl. 2) No diagnosis of interest  Incl. 3) Diagnostic instrument not yet validated |
| **13** | Chwastiak L, Tsai J, Rosenheck R. Impact of health insurance status and a diagnosis of serious mental illness on whether chronically homeless individuals engage in primary care. Am J Public Health. 2012 Dec;102(12):e83–9. | Incl. 3) Assessment by self-report measure |
| **14** | Coohey C, Easton SD. Distal Stressors and Depression among Homeless Men. Health Soc Work. 2016;41(2):111–9. | Incl. 2) No diagnoses by ICD or DSM criteria |
| **15** | Dauriac-Le Masson V, Mercuel A, Guedj MJ, Douay C, Chauvin P, Laporte A, et al. Mental healthcare utilization among homeless people in the greater paris area. Int J Environ Res Public Health. 2020 Nov;17(21):1–13. | Other: *Results on this sample already included via another publication (Laporte et al. 2018)* |
| **16** | Dealberto M-JCC, Middlebro A, Farrell S. Symptoms of schizophrenia and psychosis according to foreign birth in a Canadian sample of homeless persons. Psychiatr Serv. 2011 Oct 1;62(10):1187–93. | Excl. 2) Selected subpopulation – only homeless individuals referred for psychiatric symptoms |
| **17** | Diaz Vickery K, Guzman-Corrales L, Owen R, Soderlund D, Shimotsu S, Clifford P, et al. Medicaid expansion and mental health: A Minnesota case study. Fam Syst Heal. 2016 Mar;34(1):58–63. | Incl. 1) Sampled participants not exclusively homeless  Incl. 2) Rates of individual diagnoses not differentiated |
| **18** | Ding K, Slate M, Yang J. History of co-occurring disorders and current mental health status among homeless veterans. BMC Public Health. 2018;18(1):751. | Incl. 3) Assessment by self-report measure  Excl. 2) Selected subpopulation – only homeless veterans |
| **19** | Dittrich LO, Hava P. Determinants of health among homeless population in the Czech Republic--an empirical study. Cent Eur J Public Health. 2009;17(4):175–8. | Incl. 2) Rates of individual diagnoses not differentiated  Incl. 3) Assessment by self-report measure |
| **20** | Dunne E, Duggan M, O’Mahony J. Mental health services for homeless: Patient profile and factors associated with suicide and homicide. Ir Med J. 2012;105(3):71–4. | Excl. 2) Selected subpopulation – only homeless individuals attending mental health services |
| **21** | Edens EL, Mares AS, Rosenheck RA. Chronically homeless women report high rates of substance use problems equivalent to chronically homeless men. Women’s Heal Issues. 2011 Sep;21(5):383–9. | Incl. 3) Assessment by self-report measure |
| **22** | Feodor Nilsson S, Hjorthoj CR, Erlangsen A, Nordentoft M. Suicide and unintentional injury mortality among homeless people: a Danish nationwide register-based cohort study. Eur J Public Health. 2014;24(1):50–6. | Incl. 4) Registry study format does not provide point prevalence or 12-months-prevalence rates  Excl. 2) Selected subpopulation – only homeless individuals treated at psychiatric institutions |
| **23** | Fox AM, Mulvey P, Katz CM, Shafer MS. Untangling the relationship between mental health and homelessness among a sample of arrestees. Crime Delinq. 2016 May;62(5):592–613. | Incl. 1) Sampled participants not exclusively homeless  Incl. 3) Assessment by self-report measure |
| **24** | Ganesh A, Campbell DJT, Hurley J, Patten S. High positive psychiatric screening rates in an urban homeless population. Can J Psychiatry. 2013 Jun;58(6):353–60. | Incl. 2) No diagnoses by ICD or DSM criteria |
| **25** | Gentil L, Grenier G, Fleury M-J. Determinants of suicidal ideation and suicide attempt among former and currently homeless individuals. Soc Psychiatry Psychiatr Epidemiol. 2020;1–11. | Incl. 1) Sampled participants not exclusively homeless |
| **26** | Ghose T, Gordon AJ, Metraux S, Justice AC. Mental illness and homelessness among veterans. Psychiatr Serv. 2011 Dec 1;62(12):1514–5. | Incl. 1) Sampled participants not exclusively homeless |
| **27** | Goldstein G, Luther JF, Haas GL, Gordon AJ, Appelt C. Comorbidity between psychiatric and general medical disorders in homeless veterans. Psychiatr Q. 2009 Dec;80(4):199–212. | Excl. 2) Selected subpopulation – only homeless veterans |
| **28** | Grinman MN, Chiu S, Redelmeier DA, Levinson W, Kiss A, Tolomiczenko G, et al. Drug problems among homeless individuals in Toronto, Canada: prevalence, drugs of choice, and relation to health status. BMC Public Health. 2010;10:94. | Incl. 2) No diagnoses by ICD or DSM criteria |
| **29** | Guenzel N, Ivanich J, Habecker P, Struwe L, Hinrichsen S. Mental health, stigma, and barriers to care in a Midwestern sample of homeless individuals. J Soc Distress Homelessness. 2020;29(2):102–9. | Incl. 2) No diagnoses by ICD or DSM criteria  Incl. 3) Assessment by self-report measure |
| **30** | Guillen AI, Marin C, Panadero S, Vazquez JJ, Guillén AI, Marín C, et al. Substance use, stressful life events and mental health: a longitudinal study among homeless women in Madrid (Spain). Addict Behav. 2020;103:106246. | Incl. 2) No diagnoses by ICD or DSM criteria |
| **31** | Henry J-M, Boyer L, Belzeaux R, Baumstarck-Barrau K, Samuelian J-C. Mental disorders among homeless people admitted to a French psychiatric emergency service. Psychiatr Serv. 2010 Mar;61(3):264–71. | Incl. 4) Retrospective design does not provide point prevalence or 12-months-prevalence rates  Excl. 2) Selected subpopulation – only homeless individuals referred to a psychiatric ward |
| **32** | Hewett N, Hiley A, Gray J. Morbidity trends in the population of a specialised homeless primary care service. Br J Gen Pract. 2011;61(584):200–2. | Incl. 4) Retrospective design does not provide point prevalence or 12-months-prevalence rates  Excl. 2) Selected subpopulation – only homeless individuals referred to a specialized care service |
| **33** | Hwang SW, Gogosis E, Chambers C, Dunn JR, Hoch JS, Aubry T. Health status, quality of life, residential stability, substance use, and health care utilization among adults applying to a supportive housing program. J Urban Health. 2011;88(6):1076–90. | Incl. 1) Sampled participants not exclusively homeless  Incl. 2) No diagnoses by ICD or DSM criteria |
| **34** | Inouye SK, O’Connell JJ, Puelle MR. A piece of my mind. Falling off the edge. JAMA. 2013;309(5):451–2. | Other: *case series* |
| **35** | Irwin J, LaGory M, Ritchey F, Fitzpatrick K. Social assets and mental distress among the homeless: Exploring the roles of social support and other forms of social capital on depression. Soc Sci Med. 2008 Dec;67(12):1935–43. | Incl. 2) No diagnoses by ICD or DSM criteria |
| **36** | Ito K, Morikawa S, Okamura T, Shimokado K. Factors associated with mental well-being of homeless people in Japan. Psychiatry Clin Neurosci. 2014;68(2):145–53. | Incl. 2) No diagnoses by ICD or DSM criteria |
| **37** | Iwundu CN, Chen T-A, Edereka-Great K, Businelle MS, Kendzor DE, Reitzel LR. Mental illness and youth-onset homelessness: A retrospective study among adults experiencing homelessness. Int J Environ Res Public Health. 2020 Nov;17(22):1–13. | Incl. 3) Assessment by self-report measure  Incl. 4) Only lifetime prevalence rates reported |
| **38** | Keogh C, O’Brien KK, Hoban A, O’Carroll A, Fahey T. Health and use of health services of people who are homeless and at risk of homelessness who receive free primary health care in Dublin. BMC Health Serv Res. 2015;15:58. | Incl. 1) Sampled participants not exclusively homeless  Incl. 2) No diagnoses by ICD or DSM criteria |
| **39** | Kim MM, Ford JD, Howard DL, Bradford DW. Assessing trauma, substance abuse, and mental health in a sample of homeless men. Health Soc Work. 2010 Feb;35(1):39–48. | Incl. 2) No diagnoses by ICD or DSM criteria  Incl. 3) Assessment by self-report measure |
| **40** | Klineberg E, Vatiliotis V, Kang M, Medlow S, Sullivan L, Cummings M, et al. Health status of marginalised young people in unstable accommodation. J Paediatr Child Health. 2017;53(10):995–9. | Incl. 1) Sampled participants not exclusively homeless  Incl. 2) No diagnoses by ICD or DSM criteria  Excl. 2) Selected subpopulation – only homeless individuals aged 12-25 years presenting to a specialized health service |
| **41** | Lebrun‐Harris LA, Baggett TP, Jenkins DM, Sripipatana A, Sharma R, Hayashi AS, et al. Health status and health care experiences among homeless patients in federally supported health centers: Findings from the 2009 Patient Survey. Health Serv Res. 2013 Jun;48(3):992–1017. | Incl. 3) Assessment by self-report measure  Incl. 4) Only lifetime prevalence rates reported  Excl. 2) Selected subpopulation – only homeless individuals presenting to public health centres |
| **42** | Lee CM, Mangurian C, Tieu L, Ponath C, Guzman D, Kushel M. Childhood adversities associated with poor adult mental health outcomes in older homeless adults: Results from the HOPE HOME study. Am J Geriatr Psychiatry. 2017 Feb;25(2):107–17. | Incl. 2) No diagnoses by ICD or DSM criteria  Excl. 2) Selected subpopulation – only homeless individuals aged over 50 |
| **43** | Lee KH, Jun JS, Kim YJ, Roh S, Moon SS, Bukonda N, et al. Mental health, substance abuse, and suicide among homeless adults. J Evidence-Informed Soc Work. 2017 Jul;14(4):229–42. | Incl. 2) No diagnoses by ICD or DSM criteria |
| **44** | Levitt AJ, Culhane DP, DeGenova J, O’Quinn P, Bainbridge J. Health and social characteristics of homeless adults in Manhattan who were chronically or not chronically unsheltered. Psychiatr Serv. 2009 Jul;60(7):978–81. | Incl. 3) Assessment by self-report measure  Incl. 4) Only lifetime prevalence rates reported |
| **45** | Levorato S, Bocci G, Troiano G, Messina G, Nante N. Health status of homeless persons: a pilot study in the Padua municipal dorm. Ann Ig. 2017;29(1):54–62. | Incl. 2) No diagnoses by ICD or DSM criteria  Incl. 3) Assessment by self-report measure |
| **46** | Mackelprang JL, Klest B, Najmabadi SJ, Valley-Gray S, Gonzalez EA, Cash RE (Gene). Betrayal trauma among homeless adults: Associations with revictimization, psychological well-being, and health. J Interpers Violence. 2014 Apr;29(6):1028–49. | Incl. 1) Sampled participants not exclusively currently homeless  Incl. 2) No diagnoses by ICD or DSM criteria |
| **47** | Manning RM. Recovery in homelessness: The influence of choice and mastery on physical health, psychiatric symptoms, alcohol and drug use, and community integration. Psychiatr Rehabil J. 2019;42(2):147–57. | Incl. 2) No diagnoses by ICD or DSM criteria |
| **48** | Martin-Baena D, Montero-Pinar I, Fuertes-Lanzuela MI. Factors Associated to Health Needs from Homeless Perspective in Spain. Int J Stud Nurs. 2016;1(1):61. | Incl. 2) No diagnoses by ICD or DSM criteria  Incl. 3) Assessment by self-report measure |
| **49** | Matejkowski J, Lee S, Henwood B, Lukens J. Perceptions of health intervene in the relationship between psychiatric symptoms and quality of life for individuals in supportive housing. J Behav Health Serv Res. 2013;40(4):469–75. | Incl. 2) No diagnoses by ICD or DSM criteria |
| **50** | Meacham MC, Bahorik AL, Shumway M, Marguez C. Condomless Sex and Psychiatric Comorbidity in the Context of Constrained Survival Choices: A Longitudinal Study Among Homeless and Unstably Housed Women. AIDS Behav. 2019;23(3):802–12. | Incl. 1) Sampled participants not exclusively homeless |
| **51** | Montiel JM, Bartholomeu D, Carvalho L de F, Pessotto F. Assessment of Personality Disorders in Street Dwellers. Psicol Ciência e Profissão. 2015;35(2):488–502. | Incl. 5) Study location not a World Bank High-Income Country (Brazil) |
| **52** | Muldoon KA, Duff PK, Fielden S, Anema A. Food insufficiency is associated with psychiatric morbidity in a nationally representative study of mental illness among food insecure Canadians. Soc Psychiatry Psychiatr Epidemiol. 2013;48(5):795–803. | Incl. 1) Sampled participants not exclusively homeless  Incl. 3) Assessment by self-report measure |
| **53** | Nagy-Borsy E, Vági Z, Skerlecz P, Szeitl B, Kiss I, Rákosy Z. Health status and health behaviour of the Hungarian homeless people. Arch Public Heal. 2021;79(1):1–12. | Incl. 3) Assessment by self-report measure |
| **54** | Navarro-Lashayas MA, Eiroa-Orosa FJ. Substance use and psychological distress is related with accommodation status among homeless immigrants. Am J Orthopsychiatry. 2017;87(1):23–33. | Incl. 2) No diagnoses by ICD or DSM criteria  Excl. 2) Selected subpopulation – only homeless immigrants |
| **55** | Nayak RB, Patil S, Patil N, Chate SS, Koparde VA. Psychiatric morbidity among inmates of center for destitutes: A cross-sectional study. J Sci Soc. 2015;42(2):92. | Incl. 5) Study location not a World Bank High-Income Country (India) |
| **56** | Nielsen SF, Hjorthøj CR, Erlangsen A, Nordentoft M, Erlangsen A. Psychiatric disorders and mortality among people in homeless shelters in Denmark: A nationwide register-based cohort study. Lancet. 2011 Jun 25;377(9784):2205–14. | Incl. 4) Registry study format does not provide point prevalence or 12-months-prevalence rates  Excl. 2) Selected subpopulation – only homeless individuals treated at psychiatric institutions |
| **57** | Nishio A, Horita R, Sado T, Mizutani S, Watanabe T, Uehara R, et al. Causes of homelessness prevalence: Relationship between homelessness and disability. Psychiatry Clin Neurosci. 2017 Mar;71(3):180–8. | Other: *Results on this sample already included via another publication (Nishio et al. 2015)* |
| **58** | Notaro SJ, Khan M, Kim C, Nasaruddin M, Desai K. Analysis of the health status of the homeless clients utilizing a free clinic. J Community Heal Publ Heal Promot Dis Prev. 2013 Feb;38(1):172–7. | Incl. 4) Registry study format does not provide point prevalence or 12-months-prevalence rates  Excl. 2) Selected subpopulation – only homeless individuals treated at a free health service |
| **59** | Nyamathi A, Dixon EL, Shoptaw S, Marfisee M, Gelberg L, Williams S, et al. Profile of lifetime methamphetamine use among homeless adults in Los Angeles. Drug Alcohol Depend. 2008;92(1–3):277–81. | Incl. 2) No diagnoses by ICD or DSM criteria  Excl. 2) Selected subpopulation – only homeless who were HBV-seronegative |
| **60** | Nyamathi A, Marfisee M, Slagle A, Greengold B, Liu Y, Leake B. Correlates of depressive symptoms among homeless young adults. West J Nurs Res. 2012 Feb;34(1):97–117. | Incl. 2) No diagnoses by ICD or DSM criteria  Excl. 2) Selected subpopulation – only homeless aged 15-25 years |
| **61** | O’Carroll A, O’Reilly F, O’Carroll A, O’Reilly F. Health of the homeless in Dublin: Has anything changed in the context of Ireland’s economic boom? Eur J Public Health. 2008 Oct;18(5):448–53. | Incl. 3) Assessment by self-report measure |
| **62** | Okamura T, Ito K, Morikawa S, Awata S. Suicidal behavior among homeless people in Japan. Soc Psychiatry Psychiatr Epidemiol. 2014 Apr;49(4):573–82. | Incl. 3) Assessment by self-report measure |
| **63** | Okamura T, Takeshima T, Tachimori H, Takiwaki K, Matoba Y, Awata S. Characteristics of individuals with mental illness in Tokyo homeless shelters. Psychiatr Serv. 2015 Dec 1;66(12):1290–5. | Incl. 1) Sampled participants not exclusively homeless  Incl. 2) No diagnoses by ICD or DSM criteria |
| **64** | Opalach C, Romaszko J, Jaracz M, Kuchta R, Borkowska A, Buciński A. Coping styles and alcohol dependence among homeless people. PLoS One. 2016 Sep 6;11(9). | Incl. 2) No diagnoses by ICD or DSM criteria |
| **65** | Palepu A, Gadermann A, Hubley AM, Farrell S, Gogosis E, Aubry T. Substance Use and Access to Health Care and Addiction Treatment among Homeless and Vulnerably Housed Persons in Three Canadian Cities. PLoS One. 2013;8(10):e75133. | Incl. 1) Sampled participants not exclusively homeless  Incl. 2) No diagnoses by ICD or DSM criteria |
| **66** | Palepu A, Patterson M, Strehlau V, Moniruzzamen A, Tan de Bibiana J, Frankish J, et al. Daily substance use and mental health symptoms among a cohort of homeless adults in Vancouver, British Columbia. J Urban Health. 2013;90(4):740–6. | Incl. 1) Sampled participants not exclusively homeless  Excl. 2) Selected subpopulation – only homeless individuals with a diagnosis of mental disorder |
| **67** | Panadero S, Vazquez JJ, Martin RM. Alcohol, poverty and social exclusion: Alcohol consumption among the homeless and those at risk of social exclusion in Madrid. Adicciones. 2016;29(1):33–6. | Incl. 2) No diagnoses by ICD or DSM criteria |
| **68** | Patterson AA, Holden RR. Psychache and suicide ideation among men who are homeless: a test of Shneidman’s model. Suicide Life-Threatening Behav. 2012 Apr;42(2):147–56. | Incl. 2) No diagnoses by ICD or DSM criteria |
| **69** | Pluck G, Lee K-H, Lauder HE, Fox JM, Spence SA, Parks RW. Time perspective, depression, and substance misuse among the homeless. J Psychol Interdiscip Appl. 2008 Mar;142(2):159–68. | Incl. 2) No diagnoses by ICD or DSM criteria  Incl. 3) Assessment by self-report measure |
| **70** | Powell K, Maguire N. Paranoia and maladaptive behaviours in homelessness: The mediating role of emotion regulation. Psychol Psychother. 2018;91(3):363–79. | Incl. 2) No diagnoses by ICD or DSM criteria |
| **71** | Prinsloo B, Parr C, Fenton J. Mental illness among the homeless: Prevalence study in a Dublin homeless hostel. Ir J Psychol Med. 2012 Apr;29(1):22–6. | Excl. 1) Response rate at 39% |
| **72** | Puig VJ. Salud mental de las personas sin hogar. Estudio observacional de campaña en Ciutat Vella, barcelona. Salut i poblacions vulnerables. 2019;26(2):63. | Incl. 1) Sampled participants not exclusively homeless |
| **73** | Reitzel LR, Chinamuthevi S, Daundasekara SS, Hernandez DC, Chen T-A, Harkara Y, et al. Association of Problematic Alcohol Use and Food Insecurity among Homeless Men and Women. Int J Environ Res Public Health. 2020;17(10):3631. | Incl. 3) Assessment by self-report measure |
| **74** | Rodríguez-Pellejero JM, Núñez JL, Hernández D. Perfiles de personalidad y síndromes clínicos en personas sin hogar. Rev Psicopatología y Psicol Clínica. 2017 Dec;22(3):197–206. | Incl. 4) Registry study format does not provide point prevalence or 12-months-prevalence rates  Excl. 2) Selected subpopulation – only homeless individuals who had been homeless for at least a year |
| **75** | Romaszko J, Kuchta R, Opalach C, Bertrand-Bucinska A, Romaszko AM, Giergielewicz-Januszko B, et al. Socioeconomic Characteristics, Health Risk Factors and Alcohol Consumption among the Homeless in North-Eastern Part of Poland. Cent Eur J Public Health. 2017;25(1):29–34. | Incl. 2) No diagnoses by ICD or DSM criteria |
| **76** | Rondet C, Cornet P, Kaoutar B, Lebas J, Chauvin P. Depression prevalence and primary care among vulnerable patients at a free outpatient clinic in Paris, France, in 2010: results of a cross-sectional survey. BMC Fam Pract. 2013;14:151. | Incl. 1) Sampled participants not exclusively homeless |
| **77** | Roze M, Vandentorren S. Mental health of mothers and children of homeless families in Ile de France. Results of the ENFAMS survey. Neuropsychiatr Enfance Adolesc. 2019; | Excl. 2) Selected subpopulation – only homeless individuals in a certain family constellation |
| **78** | Roze M, Vandentorren S, van der waerden J, Melchior M. Factors associated with depression among homeless mothers. Results of the ENFAMS survey. J Affect Disord. 2018 Mar 15;229:314–21. | Excl. 2) Selected subpopulation – only homeless individuals in a certain family constellation |
| **79** | Salavera C, Tricas JM, Lucha O. Personality disorders and psychosocial problems in a group of participants to therapeutic processes for people with severe social disabilities. BMC Psychiatry. 2011;192. | Other: *Multiple methodological issues detected in quality assessment* |
| **80** | Sarajlija M, Jugovic A, Zivaljevic D, Merdovic B, Sarajlija A. Assessment of health status and quality of life of homeless persons in Belgrade, Serbia. Vojnosanit Pregl. 2014;71(2):167–74. | Incl. 3) Assessment by self-report measure  Incl. 4) Only lifetime prevalence rates reported |
| **81** | Schiltz L, Ciccarello A, Ricci-Boyer L, Schiltz J. Great precariousness, psycho-trauma, narcissistic suffering: Results of action-research based on an integrated quantitative and qualitative research methodology. Ann Med Psychol (Paris). 2014 Sep;172(7):513–8. | Incl. 1) Sampled participants not exclusively homeless  Incl. 2) No diagnoses by ICD or DSM criteria |
| **82** | Sharman S, Dreyer J, Aitken M, Clark L, Bowden-Jones H. Rates of problematic gambling in a British homeless sample: a preliminary study. J Gambl Stud. 2015;31(2):525–32. | Incl. 2) No diagnosis of interest |
| **83** | Sharman S, Dreyer J, Clark L, Bowden-Jones H. Down and out in London: Addictive behaviors in homelessness. J Behav Addict. 2016 Jun;5(2):318–24. | Incl. 2) No diagnosis of interest |
| **84** | Siqueira GR de, Vasconcelos DT de, Duarte GC, Arruda IC de, Costa JAS da, Cardoso R de O. Analysis of depression in elderly living in the shelter “Christ the Redeemer”, applying the Scale of Geriatric Depression (SGD). Cienc e Saude Coletiva. 2009;14(1):253–9. | Excl. 2) Selected subpopulation – only homeless individuals aged over 60 |
| **85** | Spicer B, Smith DI, Conroy E, Flatau PR, Burns L. Mental illness and housing outcomes among a sample of homeless men in an Australian urban centre. Aust N Z J Psychiatry. 2015 May;49(5):471–80. | Incl. 3) Assessment by self-report measure  Incl. 4) Only lifetime prevalence rates reported |
| **86** | Stenius-Ayoade A, Haaramo P, Erkkila E, Marola N, Nousiainen K, Wahlbeck K, et al. Mental disorders and the use of primary health care services among homeless shelter users in the Helsinki metropolitan area, Finland. BMC Health Serv Res. 2017;17(1):428. | Incl. 4) Registry study format does not provide point prevalence or 12-months-prevalence rates  Excl. 2) Selected subpopulation – only homeless individuals who presented to primary health care services |
| **87** | Strehlau V, Torchall I, Li K, Schuetz C, Kraus M. Mental health, concurrent disorders, and health care utilization in homeless women. J Psychiatr Pract. 2012 Sep;18(5):349–60. | Other: *Results on this sample already included via another publication (Krausz et al. 2013)* |
| **88** | Stringfellow EJ, Kim TW, Gordon AJ, Pollio DE, Grucza RA, Austin EL, et al. Substance use among persons with homeless experience in primary care. Subst Abus. 2016 Oct;37(4):534–41. | Incl. 2) No diagnoses by ICD or DSM criteria |
| **89** | Sun S, Irestig R, Burström B, Beijer U, Burström K. Health-related quality of life (EQ-5D) among homeless persons compared to a general population sample in Stockholm County, 2006. Scand J Public Health. 2012 Mar;40(2):115–25. | Incl. 2) No diagnoses by ICD or DSM criteria |
| **90** | Taylor KM, Sharpe L. Trauma and post-traumatic stress disorder among homeless adults in Sydney. Aust N Z J Psychiatry. 2008 Mar;42(3):206–13. | Incl. 2) No diagnosis of interest |
| **91** | Taylor-Seehafer M, Jacobvitz D, Steiker LH. Patterns of attachment organization, social connectedness, and substance use in a sample of older homeless adolescents: Preliminary findings. Fam Community Heal J Heal Promot Maint. 2008 Jan;31(Suppl1):S81–8. | Incl. 2) No diagnoses by ICD or DSM criteria  Excl. 2) Selected subpopulation – only homeless individuals aged 16-23 years |
| **92** | Termorshuizen F, van Bergen APL, Smit RBJ, Smeets HM, van Ameijden EJC. Mortality and psychiatric disorders among public mental health care clients in Utrecht: A register-based cohort study. Int J Soc Psychiatry. 2014 Aug;60(5):426–35. | Incl. 1) Sampled participants not exclusively homeless  Incl. 4) Registry study format does not provide point prevalence or 12-months-prevalence rates  Excl. 2) Selected subpopulation – only homeless individuals who presented to public health care services |
| **93** | Tompsett CJ, Domoff SE, Toro PA. Peer substance use and homelessness predicting substance abuse from adolescence through early adulthood. Am J Community Psychol. 2013 Jun;51(3–4):520–9. | Incl. 2) No diagnoses by ICD or DSM criteria  Excl. 2) Selected subpopulation – only homeless individuals aged 13-25 years |
| **94** | Topp L, Hudson SL, Maher L. Mental health symptoms among street-based psychostimulant injectors in Sydney’s Kings Cross. Subst Use Misuse. 2010 May;45(7–8):1180–200. | Incl. 2) No diagnoses by ICD or DSM criteria  Excl. 2) Selected subpopulation – only homeless individuals injecting psychostimulants |
| **95** | Torchalla I, Strehlau V, Li K, Aube Linden I, Noel F, Krausz M. Posttraumatic stress disorder and substance use disorder comorbidity in homeless adults: Prevalence, correlates, and sex differences. Psychol Addict Behav. 2014 Jun;28(2):443–52. | Other: *Results on this sample already included via another publication (Krausz et al. 2013)* |
| **96** | Torchalla I, Strehlau V, Li K, Krausz M. Substance use and predictors of substance dependence in homeless women. Drug Alcohol Depend. 2011 Nov 1;118(2–3):173–9. | Other: *Results on this sample already included via another publication (Krausz et al. 2013)* |
| **97** | Toro PA, Hobden KL, Durham KW, Oko-Riebau M, Bokszczanin A. Comparing the Characteristics of Homeless Adults in Poland and the United States. Am J Community Psychol. 2014 Mar;53(1–2):134–45. | Incl. 4) Only lifetime prevalence rates reported |
| **98** | Tsai AC, Weiser SD, Dilworth SE, Shumway M, Riley ED. Violent Victimization, Mental Health, and Service Utilization Outcomes in a Cohort of Homeless and Unstably Housed Women Living With or at Risk of Becoming Infected With HIV. Am J Epidemiol. 2015;181(10):817–26. | Incl. 1) Sampled participants not exclusively homeless  Excl. 2) HIV-positive women deliberately oversampled |
| **99** | Tucker JS, Wenzel SL, Golinelli D, Zhou A, Green HDJ. Predictors of substance abuse treatment need and receipt among homeless women. J Subst Abuse Treat. 2011 Apr;40(3):287–94. | Excl. 2) Selected subpopulation – only homeless individuals screened positive for past-year substance abuse |
| **100** | Upshur CC, Jenkins D, Weinreb L, Gelberg L, Orvek EA. Prevalence and predictors of substance use disorders among homeless women seeking primary care: An 11 site survey. Am J Addict. 2017 Jul 5; | Incl. 3) Assessment by self-report measure |
| **101** | Van Straaten B, Rodenburg G, Van der Laan J, Boersma SN, Wolf JRLM, Van de Mheen D. Substance use among Dutch homeless people, a follow-up study: Prevalence, pattern and housing status. Eur J Public Health. 2016 Feb 1;26(1):111–6. | Incl. 1) Sampled participants not exclusively homeless |
| **102** | Wagner J, Diehl K, Mutsch L, Burkert N, Löffler W, Freidl W. Health status and utilisation of the healthcare system by homeless and non‐homeless people in Vienna. Health Soc Care Community. 2014 May;22(3):300–7. | Incl. 3) Assessment by self-report measure |
| **103** | Welsh KJ, Patel CB, Fernando RC, Torres JD, Medrek SK, Schnapp WB, et al. Prevalence of bipolar disorder and schizophrenia in Houston Outreach Medicine, Education, and Social Services (HOMES) clinic patients: Implications for student-managed clinics for underserved populations. Acad Med. 2012 May;87(5):656–61. | Incl. 4) Only lifetime prevalence rates reported  Excl. 2) Selected subpopulation – only homeless individuals admitted to a student-managed free clinic |
| **104** | Winetrobe H, Wenzel S, Rhoades H, Henwood B, Rice E, Harris T. Differences in health and social support between homeless men and women entering permanent supportive housing. Women’s Heal Issues. 2017 May;27(3):286–93. | Incl. 2) No diagnoses by ICD or DSM criteria |
| **105** | Zhang L, Norena M, Gadermann A, Hubley A, Russell L, Aubry T, et al. Concurrent Disorder and Health Care Utilization among Homeless and Vulnerably Housed Persons in Canada. J Dual Diagn. 2018;1–36. | Incl. 3) Assessment by self-report measure  Incl. 4) Only lifetime prevalence rates reported |
| Incl. = Inclusion Criterion; Excl. = Exclusion Criterion | | |
| Inclusion criterion 1): Homelessness status of study participants was validated by an operationalized definition or a sampling method that specifically targeted homeless population  Inclusion criterion 2): Standardized criteria for psychiatric disorders based on the International Classification of Diseases (ICD) or Diagnostic and Statistical Manual of Mental Disorders (DSM) were applied  Inclusion criterion 3): Psychiatric diagnoses were made by clinical examination or interviews using validated semi-structured diagnostic instruments  Inclusion criterion 4): For any psychiatric disorders except for personality disorders (where lifetime rates were used), prevalence rates were reported within 12 months  Inclusion criterion 5): Study location was a high-income country according to the classification of the World Bank  Exclusion criterion 1): Response rate below 50%  Exclusion criterion 2): Only selected sub-populations sampled | | |
