# Supplementary material for: The prevalence of mental disorders among homeless people in high-income countries: An updated systematic review and meta-regression analysis
Source: PLoS Med. 2021 Aug 23;18(8):e1003750. doi: 10.1371/journal.pmed.1003750 (PMC8423293; doi:10.1371/journal.pmed.1003750)
Supplement: S4 Table — (DOCX) [file pmed.1003750.s004.docx]

| **S4 Table. Study Characteristics** | | | | | | | | | | | | |
| --- | --- | --- | --- | --- | --- | --- | --- | --- | --- | --- | --- | --- |
| *Study* | *Location* | *Period of Data Collection* | *Definition of Homelessness* | *Sampling Method* | *Recruitment Strategy* | *Participation Rate* | *Sample Size* | *Mean Age (years)* | *Sex Ratio (female/all)* | *Instrument* | *Diagnostic Criteria* | *Assessment by Mental Health Professionals* |
| Adams, 1996 | London, UK | 1991 | Residents of a hotel for homeless women | Data collected on all residents | Residents of a single shelter for the homeless | 100% | 64 | n.r. | 100% | SCID-PD | DSM-III-R | Yes |
| Bassuk, 1984 | Boston, MA, USA | 1983 | Residents of a shelter for the homeless | Data collected on all residents | Residents of a single shelter for the homeless | 100% | 78 | n.r. | 17% | Clinical Interview | DSM-III | Yes |
| Bäuml, 2017 | Munich, Germany | 2010-2012 | Residents of Homeless Shelters | Representative, randomized | Residents of all the city’s homeless shelters | 56% | 223 | 48.1 | 22% | SCID-I, SCID-II | ICD-10 | n.r. |
| Briner, 2017 | Zurich, Switzerland | 2012-2013 | Modified FEANTSA criteria fulfilled for at least 30 days | Data collected on all individuals registered | Registered clients of all communal care programs for the homeless | 74% | 338 | 45.1 | 25% | M.I.N.I. 6.0, SCID (sections B, C) | ICD-10 | Yes |
| Conolly, 2008 | New York City, NY, USA | n.r. | Clients of homeless drop-in centres | Convenience Sampling (first 60 to sign up) | Clients of two New York City homeless drop-in centres | n.a. | 60 | 41.3 | 30% | SCID-II-PQ | DSM-IV | Yes |
| Doutney, 1985 | Sydney, Australia | 1983 | Residents of a hotel for homeless men | Randomized | Residents of a single shelter for the homeless | 77% | 91 | n.r. | 0 | Clinical interview | DSM-III | Yes |
| Dufeu, 1996 | Berlin, Germany | 1993-1994 | Currently lacking private residence, spending nights at shelters, at friends or in places not intended for habitation | Randomized | Clients of a single meal service for the homeless | 85% | 72 | 40.5 | 0 | Clinical interview, CIDI (sections I, L) | ICD-10 (alcohol dependence), ICD-9 (any other diagnosis) | n.r. |
| Eikelmann, 1992 | Münster, Germany | 1990 | Residents of a night shelter for men | Data collected on all eligible individuals | Residents of a single shelter for the homeless staying for >3 months | 100% | 52 | 45 | 0 | Semi-structured interview (not specified) | ICD-10 | Yes |
| Fichter, 2001 | Munich, Germany | 1994-1996 | In the past 30 days, having slept at homes of friends and relatives or in places not intended for habitation or (at least one night) at a homeless night shelter | Representative, randomized | 1) clients of homeless shelters, 2) clients of meal services, 3) individuals who used neither | 88% | 265 | 44.7 | 0 | SCID-I | DSM-IV | Yes |
| Fischer, 1986 | Baltimore, MD, USA | 1981-1982 | "persons living in settings not readily classifiable as households or institutions" | Randomized | Individuals in walking distance of all missions in a geographically defined catchment area | 98% | 51 | n.r. | 6% | DIS | DSM-III | No |
| Freeman, 1979 | Toronto, Canada | n.r. | Men attending missions for the homeless | Randomized | Residents of a single mission for the homeless | 84% | 60 | n.r. | 0 | PSE (9^th^ Edition) | DSM (Edition not specified) | Yes |
| Geddes, 1994 | Edinburgh, UK | 1992 | Individuals lacking any kind of shelter (“roofless”) in a particular night | Data collected on all eligible individuals | All rough sleeping persons in one night in a defined city area | n.r. | 65 | 40 | 15% | PSE (screening version) | ICD-9 | No |
| Gill, 2003 | Multiple sites across the UK | 1994 | Users of public services for the homeless | Randomized | Residents of hostels, private sector leased accommodations, night shelters and day care centers | 93% | 738 | n.r. | n.r. | CIS-R | ICD-10 | Yes |
| Greifenhagen, 1997 | Munich, Germany | n.r. | Lacking private residence for at least 30 days, during which having slept rough at least once or changed accommodation at least 5 times | Representative, Randomized | Individuals in contact with street-working teams | 89% | 32 | 35.5 | 100% | DIS | DSM-III | Yes |
| Haugland, 1997 | Westchester County, NY, USA | 1993 | Applicants for an emergency homeless shelter | Data collected on all applicants over a 12-week-period | all homeless persons who requested emergency shelter in the county | 77% | 201 | 37 | 10% | Clinical Interview | DSM-III-R | Yes |
| Herrmann, 1989 | Melbourne, Australia | 1987 | Residents in accommodation for the homeless | Randomized | Residents of homeless shelters and special accommodation houses | 66% | 382 | n.r. | 18% | SCID-R | DSM-III-R | Yes |
| Hynes, 2018 | Dublin, Ireland | 2011 | ETHOS criteria - only entrenched rough sleepers (for at least 1 year) | Data collected on all identified Individuals | Individuals identified by a public support agency | 72% | 16 | 45 | 14% | Clinical Interview | ICD-10 | Yes |
| Kershaw, 2003 | Glasgow, UK | 1999 | Residents of homeless hostels and rough sleepers | Representative, Randomized | Clients of hostels and drop-in centres | 78% | 225 | n.r. | 14% | CIS-R | ICD-10 | No |
| Koegel, 1988 | Los Angeles, CA, USA | n.r. | No private residence in the past 30 days | Representative, Randomized | Individuals in shelters, at meal services or congregating areas | 85% | 374 | n.r. | 4% | DIS | DSM-III | n.r. |
| Kovess, 1999 | Paris, France | 1996 | Individuals spending nights in shelters, places inappropriate for habitation or with friends and relatives on a non-permanent basis | Representative, Randomized | Clients of night shelters or meal services | 64% | 715 | n.r. | 15% | CIDI/CIDIS | ICD-10 | Yes |
| Krausz, 2013 | Three Sites across British Columbia, Canada | 2009 | Individuals currently living in places not designed for habitation or in a homeless shelter | Purposeful, non-randomized sampling with emphasis on young, female, and indigenous participants | Selected shelters, drop-in centres, and public hot spots | n.r. | 489 | 37.9 | 40% | M.I.N.I.-PLUS | DSM-IV | Yes |
| Längle, 2005 | Tübingen, Germany | 2002-2003 | Section 72 of the Federal German Social Assistance Act | Data collected on all eligible individuals | individuals registered with or mediated by social services and persons directly approached on the streets | 60% | 91 | 44 | 0 | SCID-I (excluding sections I and J) | ICD-10 | Yes |
| LaPorte, 2018 | Greater Paris Area, France | 2009 | Having slept in institutional accommodation or places not intended for human habitation in the past 5 days | Representative, Randomized | Clients of public and private homeless aid programs | 71% | 840 | n.r. | 52% | M.I.N.I.-plus v.5 + non-structured clinical interview | ICD-10 | Yes |
| Madianos, 2013 | Greater Athens Area (including Piraeus), Greece | 2010-2011 | FEANTSA criteria fulfilled for the past 30 days | Randomized | Individuals identified through registers of social institutions or at rough sleeping places | 62% | 254 | 51.0 | 26% | M.I.N.I. v.5 | DSM-IV | Yes |
| Morikawa, 2011 | Tokyo, Japan | 2008-2009 | Individuals inhabiting various places not intended for accommodation | Data collected on all eligible individuals | Individuals identified through meal administration of a local non-governmental support group | 71% | 80 | 50.5 | 6% | M.I.N.I. + non-structured clinical interview | DSM-IV-TR | Yes |
| Nishio, 2015 | Nagoya, Japan | 2014 | Individuals lacking housing, including ones in shelters and temporary accommodations | n.r. | Persons mediated by a support centre for welfare consultation | n.r. | 114 | 54.0 | 7% | M.I.N.I. | DSM-IV-TR | Yes |
| North, 2009 | Saint Louis, MO, USA | 1999-2001 | No current fixed address of one’s own and having spent the previous 14 nights in a location without a personal mailing address | Randomized | Inhabitants of 12 shelters and individuals encountered in various street locations | 59% | 255 | 41.5 | 27% | CIDI | DSM-III-R | n.r. |
| Reinking, 2001 | Utrecht, Netherlands | 1998 | Having slept in night shelters or places not intended for human habitation or having received specialized day care in the past month | Randomized | Clients of day and night care facilities for the homeless | 68% | 138 | 38 | 0 | CIDI | DSM-III-R | No |
| Salize, 2001/ Salize, 2002 | Mannheim, Germany | 1997-1999 | Not regularly leasing an own room or apartment | Representative, Randomized | Residents of rehabilitative accommodations and shelters and persons living with friends/ relatives or on the streets | 80% | 102 | 40 | 14% | SCID | ICD-10 | Yes |
| Sclare, 1997 | Aberdeen, UK | 1989-1990 | Residents of a hostel for the homeless | Randomized | Residents of a single hostel for the homeless | 94% | 75 | 34.2 | 15% | PSE | n.r. | Yes |
| Smith, 1992 | Saint Louis, MO, USA | 1989-1990 | Having no stable residence and staying in a public shelter or unsheltered location without personal mailing addresses | Representative, Randomized | Individuals approached at shelters and rough sleeping hot spots | 91% | 600 | 35.9 | 0 | DIS (modified version) | DSM-III-R | Yes |
| Smith, 1993 | Saint Louis, MO, USA | 1989-1990 | Having no stable residence and staying in a public shelter or unsheltered location without personal mailing addresses | Representative, Randomized | Individuals approached at shelters and day care centers | 97% | 300 | 29 | 100% | DIS (modified version) | DSM-III-R | Yes |
| Susser, 1989 | New York City, NY, USA | 1985 | Applicants to the municipal shelter system | Convenience sampling | First time users of the municipal shelter system | 84% | 177 | n.r. | 0 | SCID-PD | DSM-III-R | No |
| Timms, 1989 | London, UK | 1986-1987 | Residents of a homeless hostel | Data collected on all permanent resident and a representative sample of new arrivals | (1) Individuals residing at a hostel for at least one year and (2) new arrivals sleeping at the hostel for the first time in at least one month | 90% | 123 | 49.5 | 0 | PSE | DSM-III | Yes |
| Torchalla, 2004 | Tübingen, Germany | 2001 | Section 72 of the Federal German Social Assistance Act | Data collected on all eligible individuals | Clients of different counselling services | 100% | 17 | 29 | 100% | SCID-I | DSM-IV | n.r. |
| Völlm, 2004 | Dortmund, Germany | 1996 | Residents of asylums/ shelters and individuals sleeping on the streets | Representative, Randomized | Clients of a representative set of specialized social services | 82% | 82 | 41.4 | 0 | AMDP, CIDI (modules I, L) | ICD-10 | Yes |
| Weller, 1987 | London, UK | 1986 | Residents of a shelter for the homeless | n.r. | Residents of a shelter for the homeless | 93% | 100 | 45.6 | 4% | PSE | n.r. | n.r. |
| Whitbeck, 2015 | Omaha, NE/ Pittsburgh, PA/ Portland, OR, USA | 2010-2011 | 1987 Steward A. McKinney Act criteria | n.r. | Individuals residing at shelters or being registered at meal services | 63% | 156 | 38.9 | 100% | CIDI | DSM-IV | n.r. |
| **Abbreviations (in the order of first appearance):**  n.r. = not reported  SCID-PD = Structured Clinical Interview for DSM-III Disorder: Psychotic Disorder version  MA = Massachusetts  SCID = Structured Clinical Interview for DSM-IV; SCID-II-PQ = Structured Clinical Interview for DSM-IV, Section II, Personality Questionnaire  FEANTSA = *Fédération Européenne d'Associations Nationales Travaillant avec les Sans-Abri* [European Federation of National Organizations Working with the Homeless]  M.I.N.I. = Mini-International Neuropsychiatric Interview  NY = New York  CIDI = World Health Organization Composite International Diagnostic Interview; CIDIS = Composite International Diagnostic Interview, simplified  MD = Maryland  DIS = Diagnostic Interview Schedule  PSE = Present State Examination  CIS-R = Clinical Interview Schedule, revised  SCID-R = Structured Clinical Interview for DSM-III-R  ETHOS = European Typology on Homelessness and Housing Exclusion  MO = Missouri  AMDP = *Psychopathologisches Interview der Arbeitsgemeinschaft Methodik und Dokumentation in der Psychiatrie* [Psychopathological Interview of the Working Group for Methodology and Documentation in Psychiatry]  NE = Nebraska  PA = Pennsylvania  OR = Oregon | | | | | | | | | | | | |
