# Supplementary material for: The prevalence of mental disorders among homeless people in high-income countries: An updated systematic review and meta-regression analysis
Source: PLoS Med. 2021 Aug 23;18(8):e1003750. doi: 10.1371/journal.pmed.1003750 (PMC8423293; doi:10.1371/journal.pmed.1003750)
Supplement: S5 Table — (DOCX) [file pmed.1003750.s005.docx]

| **S5 Table. JBI Checklist for Prevalence Studies [34]** | | | | | | | | | |
| --- | --- | --- | --- | --- | --- | --- | --- | --- | --- |
| Study | **Q1** | **Q2** | **Q3** | **Q4** | **Q5** | **Q6** | **Q7** | **Q8** | **Q9** |
| Adams, 1996 | Yes | Yes | Yes | Yes | Yes | Yes | Yes | Yes | Yes |
| Bassuk, 1984 | Yes | Yes | Yes | Yes | Yes | Yes | No | No | Unclear |
| Bäuml, 2017 | Yes | Yes | Yes | Yes | No | Yes | Unclear | No | No |
| Briner, 2017 | No | Yes | Yes | Yes | No | Yes | Unclear | No | No |
| Conolly, 2008 | Yes | No | No | Yes | No | Yes | Yes | No | n.a. |
| Doutney, 1985 | Yes | Yes | Yes | No | Yes | Yes | Yes | No | Yes |
| Dufeu, 1996 | Yes | Yes | Yes | Yes | Yes | Yes | Yes | Yes | Yes |
| Eikelmann, 1992 | Yes | Yes | Yes | Yes | Unclear | Yes | Yes | No | Unclear |
| Fichter 2001 | Yes | Yes | Yes | Yes | Yes | Yes | Yes | No | Yes |
| Fischer 1986 | Yes | Yes | Yes | Yes | Yes | Yes | Yes | No | Yes |
| Freeman 1979 | Yes | Yes | Yes | No | Yes | Yes | Yes | No | Yes |
| Geddes, 1994 | Yes | Yes | Yes | Yes | Yes | Yes | No | No | Unclear |
| Gill, 2003 | Yes | Yes | Yes | No | Yes | Yes | Yes | No | Yes |
| Greifenhagen, 1997 | Yes | Yes | No | Yes | Yes | Yes | Yes | No | Yes |
| Haugland, 1997 | No | No | Yes | Yes | Yes | Yes | No | No | Yes |
| Herrman, 1989 | Yes | Yes | Yes | Yes | No | Yes | Yes | No | No |
| Hynes, 2018 | Yes | Yes | Yes | Yes | No | Yes | No | Yes | Yes |
| Kershaw, 2003 | Yes | Yes | Yes | No | Unclear | No | No | No | Yes |
| Koegel 1988 | Yes | Yes | Yes | No | Yes | Yes | Yes | No | Yes |
| Kovess, 1999 | Yes | Yes | Yes | Yes | Yes | Yes | Yes | No | No |
| Krausz, 2013 | Yes | No | Yes | Yes | Unclear | Yes | Yes | No | Unclear |
| Längle, 2005 | Yes | Yes | Yes | Yes | No | Yes | Yes | No | No |
| LaPorte, 2018 | Yes | Yes | Yes | No | Unclear | Yes | Yes | Yes | No |
| Madianos, 2013 | Yes | No | Yes | Yes | Unclear | Yes | Yes | No | No |
| Morikawa, 2011 | Yes | Yes | No | Yes | No | Yes | Yes | No | No |
| Nishio, 2015 | Yes | Unclear | No | Yes | Unclear | Unclear | Yes | Yes | Unclear |
| North, 2009 | Yes | Yes | Yes | Yes | Yes | Yes | Yes | No | Yes |
| Reinking, 2001 | Yes | Yes | Yes | Yes | Yes | Yes | Yes | Yes | No |
| Salize, 2001 | Yes | No | Yes | Yes | No | Yes | Yes | No | Yes |
| Salize, 2002 | Yes | No | Yes | Yes | No | Yes | Yes | No | Yes |
| Sclare, 1997 | Yes | Yes | Yes | Yes | Yes | Unclear | Yes | No | Yes |
| Smith, 1992 | Yes | Yes | Yes | Yes | Yes | Yes | Yes | No | Yes |
| Smith, 1993 | Yes | Yes | Yes | Yes | Yes | Yes | Yes | No | Yes |
| Susser, 1989 | Yes | No | Yes | No | Yes | Yes | Yes | No | Yes |
| Timms, 1989 | Yes | Yes | Yes | No | Yes | Yes | Unclear | No | Yes |
| Torchalla, 2004 | Yes | Yes | No | Yes | Unclear | Yes | Yes | No | Yes |
| Völlm, 2004 | Yes | Yes | Yes | Yes | Yes | Yes | Yes | No | No |
| Weller, 1987 | No | No | Yes | No | Yes | Yes | Yes | No | Yes |
| Whitbeck, 2015 | Yes | Unclear | Yes | Yes | No | Yes | Yes | Yes | No |
| The JBI Checklist provides quality criteria for prevalence studies in nine distinct items. The table indicates which items have been fulfilled for each included study, respectively:  Q1: Was the sample frame appropriate to address the target population? Q2: Were study participants recruited in an appropriate way? Q3: Was the sample size adequate? Q4: Were the study subjects and setting described in detail? Q5: Was data analysis conducted with sufficient coverage of the identified sample? Q6: Were valid methods used for the identification of the condition? Q7: Was the condition measured in a standard, reliable way for all participants? Q8: Was there appropriate statistical analysis? Q9: Was the response rate adequate, and if not, was the low response rate managed appropriately? | | | | | | | | | |
