# Supplementary material for: The prevalence of mental disorders among homeless people in high-income countries: An updated systematic review and meta-regression analysis
Source: PLoS Med. 2021 Aug 23;18(8):e1003750. doi: 10.1371/journal.pmed.1003750 (PMC8423293; doi:10.1371/journal.pmed.1003750)
Supplement: S6 Table — (DOCX) [file pmed.1003750.s006.docx]

| **S6 Table. Risk of Bias Tool** [35] | | | | | | | | | | |
| --- | --- | --- | --- | --- | --- | --- | --- | --- | --- | --- |
| **Study** | **Q2** | **Q3** | **Q4** | **Q5** | **Q6** | **Q7** | **Q8** | **Q9** | **Q10** | **Summary Item** |
| Adams, 1996 | Yes | Yes | Yes | Yes | Yes | Yes | Yes | Yes | Yes | Low Risk |
| Bassuk, 1984 | Yes | Yes | No | Yes | Yes | No | Yes | Yes | Yes | Moderate Risk |
| Bäuml, 2017 | Yes | Yes | No | Yes | Yes | Yes | Yes | Yes | Yes | Moderate Risk |
| Briner, 2017 | No | Yes | No | Yes | Yes | Yes | Yes | Yes | Yes | Moderate Risk |
| Conolly, 2008 | Yes | No | No | Yes | Yes | Yes | Yes | Yes | Yes | Moderate Risk |
| Doutney, 1985 | Yes | Yes | Yes | Yes | Yes | Yes | Yes | Yes | Yes | Moderate Risk |
| Dufeu, 1996 | Yes | Yes | Yes | Yes | Yes | Yes | Yes | Yes | Yes | Low Risk |
| Eikelmann, 1992 | Yes | Yes | No | Yes | Yes | Yes | Yes | Yes | Yes | Moderate Risk |
| Fichter, 2001 | Yes | Yes | Yes | Yes | Yes | Yes | Yes | Yes | No | Low Risk |
| Fischer, 1986 | Yes | Yes | Yes | Yes | Yes | Yes | Yes | Yes | Yes | Low Risk |
| Freeman, 1979 | Yes | Yes | Yes | Yes | No | Yes | Yes | Yes | Yes | Moderate Risk |
| Geddes, 1994 | Yes | Yes | No | Yes | Yes | Yes | Yes | Yes | Yes | Low Risk |
| Gill, 2003 | Yes | Yes | Yes | No | Yes | Yes | No | Yes | Yes | Moderate Risk |
| Greifenhagen, 1997 | Yes | Yes | Yes | Yes | Yes | Yes | Yes | Yes | Yes | Low Risk |
| Haugland, 1997 | No | No | Yes | Yes | Yes | No | Yes | Yes | Yes | Moderate Risk |
| Herrman, 1989 | Yes | Yes | No | Yes | Yes | Yes | Yes | Yes | Yes | Moderate Risk |
| Hynes, 2018 | Yes | Yes | Yes | Yes | Yes | No | Yes | Yes | Yes | Low Risk |
| Kershaw, 2003 | Yes | Yes | Yes | Yes | No | No | Yes | Yes | Yes | Moderate Risk |
| Koegel, 1988 | Yes | Yes | Yes | Yes | Yes | Yes | Yes | Yes | Yes | Low Risk |
| Kovess, 1999 | Yes | Yes | No | Yes | Yes | Yes | Yes | Yes | Yes | Low Risk |
| Krausz, 2013 | Yes | No | No | Yes | Yes | Yes | Yes | Yes | Yes | Moderate Risk |
| Längle, 2005 | Yes | Yes | No | Yes | Yes | Yes | Yes | Yes | Yes | Moderate Risk |
| LaPorte, 2018 | Yes | Yes | No | Yes | Yes | Yes | Yes | Yes | Yes | Low Risk |
| Madianos, 2013 | Yes | Yes | Yes | Yes | Yes | Yes | Yes | Yes | Yes | Low Risk |
| Morikawa, 2011 | Yes | Yes | No | Yes | Yes | Yes | Yes | Yes | Yes | Moderate Risk |
| Nishio, 2015 | Yes | No | No | Yes | Yes | No | Yes | No | Yes | Moderate Risk |
| North, 2009 | Yes | Yes | Yes | Yes | Yes | Yes | Yes | Yes | No | Low Risk |
| Reinking, 2001 | Yes | Yes | No | Yes | Yes | Yes | Yes | Yes | Yes | Low Risk |
| Salize, 2001 | Yes | No | Yes | Yes | Yes | Yes | Yes | Yes | Yes | Moderate Risk |
| Salize, 2002 | Yes | No | Yes | Yes | Yes | Yes | Yes | Yes | Yes | Moderate Risk |
| Sclare, 1997 | Yes | Yes | Yes | Yes | No | Yes | Yes | Yes | Yes | Low Risk |
| Smith, 1992 | Yes | Yes | Yes | Yes | Yes | Yes | Yes | Yes | Yes | Low Risk |
| Smith, 1993 | Yes | Yes | Yes | Yes | Yes | Yes | Yes | Yes | Yes | Low Risk |
| Susser, 1989 | Yes | No | Yes | Yes | Yes | Yes | Yes | Yes | Yes | Moderate Risk |
| Timms, 1989 | Yes | No | Yes | Yes | Yes | Yes | Yes | Yes | Yes | Moderate Risk |
| Torchalla, 2004 | Yes | Yes | Yes | Yes | Yes | Yes | Yes | Yes | Yes | Low Risk |
| Völlm 2004 | Yes | Yes | Yes | Yes | Yes | Yes | Yes | Yes | Yes | Low Risk |
| Weller, 1987 | No | No | Yes | Yes | Yes | Yes | Yes | Yes | Yes | Moderate Risk |
| Whitbeck 2015 | Yes | No | No | Yes | Yes | Yes | Yes | Yes | Yes | Moderate Risk |
| The Risk of Bias tool provides quality criteria for prevalence studies in ten distinct items and one summary item. The table indicates which items have been fulfilled for each included study, respectively:  Q1: (*Disregarded, since not relevant for the aim of this review*)  Q2: Was the sampling frame a true or close representation of the target population?  Q3: Was some form of random selection used to select the sample, OR, was a census undertaken?  Q4: Was the likelihood of non-response bias minimal?  Q5: Were data collected directly from the subjects (as opposed to a proxy)?  Q6: Was an acceptable case definition used in the study?  Q7: Was the study instrument that measured the parameter of interest (e.g. prevalence of low back pain) shown to have reliability and validity (if necessary)? Q8: Was the same mode of data collection used for all subjects? Q9: Was the length of the shortest prevalence period for the parameter of interest appropriate?  Q10: Were the numerator(s) and denominator(s) for the parameter of interest appropriate?  Summary Item for the overall risk of study bias | | | | | | | | | | |
