# Supplementary material for: The prevalence of mental disorders among homeless people in high-income countries: An updated systematic review and meta-regression analysis
Source: PLoS Med. 2021 Aug 23;18(8):e1003750. doi: 10.1371/journal.pmed.1003750 (PMC8423293; doi:10.1371/journal.pmed.1003750)
Supplement: S7 Table — (DOCX) [file pmed.1003750.s007.docx]

| **S7 Table. Data basis for meta-analyses and meta-regression analyses** | | | | | | | | | | | | | | | | | | | |
| --- | --- | --- | --- | --- | --- | --- | --- | --- | --- | --- | --- | --- | --- | --- | --- | --- | --- | --- | --- |
| *Study* | *Sample Size* | *Cases of any current mental disorder (n)* | *Cases of schizophrenia spectrum disorders (n)* | *Cases of major depression (n)* | *Cases of bipolar disorder (n)* | *Cases of alcohol use disorder (n)* | *Cases of drug use disorder (n)* | *Cases of personality disorders (n)* | *Sex ratio (female/all)* | *Diagnostic instrument ^a^* | *Interviewer background ^b^* | *Response rate* | *Mean age (years)* | *Share of participants practicing rough sleeping* | *Sampling method ^c^* | *Mean lifetime homelessness (years)* | *Final year of assessments* | *Study location ^d^* | *Prevalence period ^e^* |
| Adams, 1996 | 64 | - | 27 | - | - | - | - | - | 1 | 1 | 1 | 1 | 44 | 0 | 0 | - | 1991 | 2 | - |
| Bassuk, 1984 | 78 | - | 28 | 3 | 4 | 23 | - | 16 | .167 | 0 | 1 | 1 | - | 0 | 0 | - | 1983 | 1 | 1 |
| Bäuml, 2017 | 223 | 208 | 23 | 36 | 8 | 86 | 56 | 119 | .215 | 1 | - | .562 | 48.1 | 0 | 1 | 5.1 | 2012 | 3 | 1 |
| Briner, 2017 | 338 | - | 85 | - | - | 140 | 206 | 51 | .246 | 1 | 1 | .735 | 45.1 | 0 | 0 | - | 2013 | 4 | 1 |
| Conolly, 2008 | 60 | - | - | - | - | - | - | 59 | .3 | 1 | 1 | - | 41.3 | - | 0 | 4.6 | 2006 | 1 | - |
| Doutney, 1985 | 91 | - | 15 | - | - | - | - | - | 0 | 0 | 1 | .771 | - | 0 | 1 | - | 1983 | 5 | 0 |
| Dufeu, 1996 | 72 | 65 | 5 | - | - | 49 | 9 | - | 0 | 1 | - | .85 | 40.5 | .347 | 1 | - | 1993 | 3 | - |
| Eikelmann, 1992 | 52 | 40 | 5 | - | - | 33 | - | 71 | 0 | 0 | - | 1 | 45 | 0 | 0 | 4.1 | 1990 | 3 | - |
| Fichter, 2001 | 265 | 195 | 17 | 16 | 5 | 162 | 14 | - | 0 | 1 | 1 | .88 | 44.7 | - | 1 | 8 | 1996 | 3 | 1 |
| Fischer, 1986 | 51 | - | 1 | - | - | - | - | - | .059 | 1 | 0 | .981 | - | 0 | 1 | - | 1982 | 1 | 1 |
| Freeman, 1979 | 60 | - | 13 | - | - | 43 | - | - | 0 | 1 | 1 | .836 | - | - | 1 | - | 1978 | 1 | 1 |
| Geddes, 1994 | 65 | - | 2 | 1 | 1 | - | - | 3 | .154 | 1 | 0 | - | 40 | 1 | 0 | - | 1992 | 2 | - |
| Gill, 2003 | 738 | - | - | - | - | 85 | - | - | - | 1 | 1 | - | - | - | - | - | 1994 | 2 | 1 |
| Greifenhagen, 1997 | 32 | - | 11 | 13 | 3 | 18 | 7 | - | 1 | 1 | 1 | .889 | 35.5 | - | 1 | 6.6 | 1996 | 3 | 0 |
| Haugland, 1997 | 201 | - | 22 | - | - | 103 | 145 | 68 | .104 | 0 | 1 | .774 | 37 | - | 0 | 3.8 | 1993 | 1 | - |
| Herrmann, 1989 | 382 | - | 67 | 25 | 4 | 85 | 36 | - | .181 | 1 | 1 | .66 | - | 0 | 1 | - | 1987 | 5 | 1 |
| Hynes, 2018 | 16 | 9 | 4 | 0 | 1 | 5 | 5 | 0 | .125 | 0 | 1 | .723 | - | 1 | 0 | - | 2011 | 4 | - |
| Kershaw, 2003 | 225 | - | - | - | - | - | 56 | - | .142 | - | - | .78 | - | 0 | - | - | 1999 | 2 | 1 |
| Koegel, 1988 | 374 | - | 38 | 51 | - | 89 | 33 | - | .04 | 1 | - | .852 | - | - | 1 | - | 1985 | 1 | 0 |
| Kovess, 1999 | 715 | - | 41 | - | 12 | 107 | 74 | - | .152 | 1 | - | .642 | - | .134 | 1 | - | 1996 | 4 | 0 |
| Krausz, 2013 | 489 | - | 73 | 111 | 59 | 182 | 343 | - | .392 | 1 | 0 | - | 37.9 | .5 | 0 | - | 2009 | 1 | 1 |
| Längle, 2005 | 91 | - | 7 | - | - | 49 | 23 | - | 0 | 1 | 1 | .603 | 44 | 0 | 0 | - | 2003 | 3 | 1 |
| LaPorte, 2018 | 840 | - | 111 | - | - | 177 | 147 | - | .521 | 1 | 1 | .71 | - | 0 | 1 | - | 2009 | 4 | - |
| Madianos, 2013 | 254 | - | 33 | 28 | 3 | 14 | 16 | - | .26 | 1 | 1 | .62 | 51 | .343 | 1 | - | 2011 | 4 | 1 |
| Morikawa, 2011 | 80 | 50 | - | - | - | 13 | - | - | .063 | 1 | 1 | .713 | 50.5 | 1 | 0 | 5.7 | 2009 | 5 | 1 |
| Nishio, 2015 | 114 | - | 5 | - | - | 16 | 0 | 4 | .07 | 1 | 1 | - | 54 | .632 | - | 3.5 | 2014 | 5 | - |
| North, 2009 | 255 | - | - | - | - | 103 | 98 | - | .267 | 1 | - | .59 | 41.5 | - | 1 | 4.4 | 2001 | 1 | 0 |
| Reinking, 2001 | 138 | - | 21 | 44 | - | 29 | 70 | - | 0 | 1 | 0 | .68 | 38 | .417 | 1 | 6 | 1998 | 4 | 0 |
| Salize, 2001/ Salize, 2002 | 102 | 70 | 7 | - | - | 47 | - | 15 | .137 | 1 | 1 | .8 | 40 | - | 0 | 9.3 | 1999 | 3 | - |
| Sclare, 1997 | 75 | - | 5 | 5 | 6 | - | - | - | .147 | 1 | 1 | - | 34.2 | 0 | 1 | - | 1990 | 2 | 1 |
| Smith, 1992 | 600 | - | 30 | 80 | 11 | 281 | 85 | 152 | 0 | 1 | 0 | .91 | 36 | .29 | 1 | 2.8 | 1989 | 1 | 0 |
| Smith, 1993 | 300 | - | 10 | 59 | 5 | 34 | 29 | 91 | 1 | 1 | - | .96 | 29 | 0 | 1 | 1.1 | 1990 | 1 | 0 |
| Susser, 1989 | 177 | - | 21 | - | - | - | - | - | 0 | 1 | 0 | .835 | - | 0 | 0 | - | 1985 | 1 | - |
| Timms, 1989 | 123 | - | 38 | - | - | - | 1 | 8 | 0 | 1 | 1 | .93 | - | 0 | 0 | - | 1987 | 2 | - |
| Torchalla, 2004 | 17 | 12 | 2 | 0 | - | 5 | - | - | 1 | 1 | - | 1 | 29 | 0 | 0 | 2.3 | 2001 | 3 | 1 |
| Völlm, 2004 | 82 | - | 4 | 13 | - | 48 | 20 | 20 | 0 | 1 | 1 | .815 | 41.4 | .537 | 1 | - | 1996 | 3 | 1 |
| Weller, 1987 | 100 | - | 23 | - | - | - | - | - | .04 | 1 | - | .926 | 45.6 | 0 | - | - | 1986 | 2 | - |
| Whitbeck, 2015 | 156 | - | - | 17 | 21 | 24 | 25 | - | 1 | 1 | - | .631 | 38.9 | - | - | - | 2011 | 1 | 1 |
| ^a^1 = semi-structured; 0 = clinical only  ^b^1 = mental-health clinician; 0 = lay interviewer  ^c^1 = randomized; 0 = non-randomized  ^d^1 = North America; 2 = United Kingdom; 3 = Germany; 4 = other European countries; 5 = Japan/Australia  ^e^1 = ≤1 month; 0 = >1 month | | | | | | | | | | | | | | | | | | | |
