# Supplementary material for: The prevalence of mental disorders among homeless people in high-income countries: An updated systematic review and meta-regression analysis
Source: PLoS Med. 2021 Aug 23;18(8):e1003750. doi: 10.1371/journal.pmed.1003750 (PMC8423293; doi:10.1371/journal.pmed.1003750)
Supplement: S8 Table — (DOCX) [file pmed.1003750.s008.docx]

| **S8 Table. Results of Univariate Meta-Regression Models:** Showing Values of β, SE(β), p-value of β, and Adjusted Coefficient of Determination (for significant models) | | | | | | | | |
| --- | --- | --- | --- | --- | --- | --- | --- | --- |
| **Study Characteristic** | **Handling of Missing Values** | **Axis-I disorders (pooled)** | **Schizophrenia Spectrum** | **Major Depression** | **Bipolar Disorder** | **Alcohol Use Disorders** | **Drug Use Disorders** | **Personality Disorders** |
| **Sample Size (continuous)** | (complete cases only) | <0.01 (<0.01) p = 0.30 | >-0.01 (<0.01)  p = 0.16 | <0.01 (<0.01) p = 0.35 | >-0.01 (<0.01) p = 0.14 | **>-0.01 (<0.01) p = 0.03**  **R^2^ = 14.5%** | >-0.01 (<0.01) p = 0.87 | <0.01 (<0.01) p = 0.87 |
| **Sex Ratio (female/all)** | Imputed Model: | - | 0.11 (0.08) p = 0.17 | - | **-** | 0.09 (0.15)  p = 0.55 | - | - |
|  | Complete Case Analysis: | -0.06 (0.07) p = 0.81 | *0.12 (0.08)*  *p = 0.13* | 0.10 (0.10) p = 0.32 | **0.16 (0.07) p = 0.03**  **R^2^ = 31.7%** | *0.08 (0.15) p = 0.59* | 0.12 (0.17) p = 0.51 | 0.03 (0.04) p = 0.50 |
| **Final Year of Assessments (continuous)** | (complete cases only) | >-0.01 (0.01) p = 0.80 | >-0.01 (<0.01) p = 0.68 | <0.01 (<0.01) p = 0.73 | <0.01 (<0.01) p = 0.17 | -0.01 (<0.01) p = 0.07 | 0.01 (0.01) p = 0.33 | <0.01 (0.01) p = 0.74 |
| **Instrument (Semi-structured vs. clinical only)** | Imputed Model: | - | -0.08 (0.07) p = 0.27 | - | - | -0.10 (0.14) p = 0.48 | -0.37 (0.19) p = 0.06 | - |
|  | Complete Case Analysis: | 0.11 (0.14) p = 0.47 | *-0.09 (0.07)*  *p = 0.18* | 0.21 (0.12) p = 0.10 | -0.06 (0.09) p = 0.55 | *-0.10 (0.14) p = 0.50* | *-0.38 (0.19) p = 0.06* | 0.14 (0.22) p = 0.53 |
| **Sampling method (Randomized vs. Non-Randomized)** | Imputed Model: | **-** | -0.08 (0.05) p = 0.09 | 0.15 (0.08)  p = 0.06 | -0.10 (0.06)  p = 0.13 | -0.01 (0.10) p = 0.95 | **-0.25 (0.11) p = 0.04**  **R^2^ = 19.3%** | 0.13 (0.19) p = 0.51 |
|  | Complete Case Analysis: | **0.23 (0.08) p = 0.03 R^2^ = 58.7%** | *-0.10 (0.05)*  *p = 0.05* | *0.16 (0.08) p = 0.06* | *-0.11 (0.05) p = 0.05* | *-0.01 (0.11) p = 0.90* | ***-0.27 (0.12) p = 0.03***  ***R^2^ = 18.8%*** | *0.16 (0.19) p = 0.43* |
| **Study Location (North America vs. Other Regions)** | (complete cases only) | - | -0.02 (0.05)  p = 0.70 | 0.04 (0.08) p = 0.63 | 0.07 (0.05) p = 0.19 | 0.06 (0.10) p = 0.57 | 0.17 (0.11) p = 0.16 | 0.30 (0.17) p = 0.10 |
| **Study Location (United Kingdom vs. Other Regions)** | (complete cases only) | - | 0.04 (0.06) p = 0.53 | -0.17 (0.11) p = 0.14 | 0.02 (0.08)  p = 0.82 | -0.07 (0.19) p = 0.70 | -0.18 (0.19) p = 0.35 | -0.33 (0.24) p = 0.20 |
| **Study Location (Germany vs. Other Regions)** | (complete cases only) | 0.22 (0.12) p = 0.10 | -0.05 (0.05) p = 0.35 | 0.03 (0.09) p = 0.77 | >-0.01 (0.07) p = 0.98 | **0.24 (0.09) p = 0.02 R^2^ = 16.9%** | -0.05 (0.13) p = 0.67 | 0.06 (0.22) p = 0.78 |
| Lines in bold font indicate significant coefficients. Lines in cursive font indicate secondary analyses. | | | | | | | | |
