# Supplementary material for: The prevalence of mental disorders among homeless people in high-income countries: An updated systematic review and meta-regression analysis
Source: PLoS Med. 2021 Aug 23;18(8):e1003750. doi: 10.1371/journal.pmed.1003750 (PMC8423293; doi:10.1371/journal.pmed.1003750)
Supplement: S9 Table — (DOCX) [file pmed.1003750.s009.docx]

| **S9 Table.** **Results of Single Factor Meta-Regression Models** Showing Values of β, SE(β), p-value of β, and Adjusted Coefficient of Determination (for significant models) – Affective Disorders (pooled) | | |
| --- | --- | --- |
| **Study Characteristic** | **Handling of Missing Values** | **Values** |
| **Sample Size (continuous)** | (complete cases only) | <0.01 (<0.01) p = 0.08 |
| **Sex Ratio (female/all)** | (complete cases only) | 0.21 (0.11) p = 0.07 |
| **Final Year of Assessments (continuous)** | (complete cases only) | **0.01 (<0.01) p < 0.01**  **R^2^ = 32.6%** |
| **Instrument (Semi-structured vs. clinical only)** | (complete cases only) | 0.16 (0.10) p = 0.13 |
| **Sampling method (Randomized vs. Non-Randomized)** | Imputed Model: | 0.03 (0.07) p = 0.62 |
|  | Complete Case Analysis: | 0.04 (0.07) p = 0.56 |
| **Study Location (North America vs. Other Regions)** | (complete cases only) | -0.02 (0.08) p = 0.84 |
| **Study Location (United Kingdom vs. Other Regions)** | (complete cases only) | -0.17 (0.09) p = 0.06 |
| **Study Location (Germany vs. Other Regions)** | (complete cases only) | >-0.01 (0.08) p = 0.98 |
