# Supplementary material for: The prevalence of mental disorders among homeless people in high-income countries: An updated systematic review and meta-regression analysis
Source: PLoS Med. 2021 Aug 23;18(8):e1003750. doi: 10.1371/journal.pmed.1003750 (PMC8423293; doi:10.1371/journal.pmed.1003750)
Supplement: S10 Table — (DOCX) [file pmed.1003750.s010.docx]

| **S10 Table.** **Results of Multiple Factor Meta-Regression** Showing Values of β, SE(β), and p-value of β– Affective Disorders (pooled) – only complete cases | |
| --- | --- |
| **Study Characteristic** | **Values** |
|  | *With R^2^ = 41.4%* |
| **Final Year of Assessments (continuous)** | **0.01 (<0.01)**  **p = 0.04** |
| **Sex Ratio (female/all)** | 0.17 (0.08) p = 0.09 |
| **Study Location (United Kingdom vs. Other Regions)** | -0.12 (0.08)  p = 0.15 |
