# Supplementary material for: The prevalence of mental disorders among homeless people in high-income countries: An updated systematic review and meta-regression analysis
Source: PLoS Med. 2021 Aug 23;18(8):e1003750. doi: 10.1371/journal.pmed.1003750 (PMC8423293; doi:10.1371/journal.pmed.1003750)
Supplement: S1 Text — Results of meta-analysis and meta-regression analysis. (DOCX) [file pmed.1003750.s011.docx]

**S1 Text. Affective Disorders**

24 studies reported estimates on prevalence rates of affective disorders (generally pool­ing Major Depression, Bipolar Affective Disorder and Dysthymia) [28,29,49,51,53-60,62-65,67,69,71,73,76-78,82]. The Random effects mean estimate was 15.9% (95% CI 11.1% - 21.4%). Prevalence estimates ranged between 0.0% and 46.9% and displayed substantial het­erogeneity (I^2^= 95% (92% - 98%). A 95% PI was estimated at 0 - 46.4%.

Univariate meta-regression models indicated that studies reported significantly higher rates of affective disorders the more recent they were (see S9 Table). Variables final year of assessments, proportion of female participants and study location UK (as opposed to other locations) got picked by automated model selection. In a multivariate meta-regression model with R^2^=41%, the change of measured prevalence rates over time was confirmed to be a significant predictor (see S10 Table).

A subgroup analysis of 12 Low Risk of Bias studies [29,49,55,58,60,62,65,67,69,71,73,78] resulted in a random effects pooled prevalence of 14.5% (95% CI 8.2% - 22.2%), with substantial heterogeneity (I^2^= 95% (87% - 98%)). There was no difference in weighted means of Low Risk of Bias and Moderate Risk of Bias studies (Q=0.31, p=0.58).
