# Supplementary material for: The prevalence of mental disorders among homeless people in high-income countries: An updated systematic review and meta-regression analysis
Source: PLoS Med. 2021 Aug 23;18(8):e1003750. doi: 10.1371/journal.pmed.1003750 (PMC8423293; doi:10.1371/journal.pmed.1003750)
Supplement: Attachment — Submitted filename: gutwinski.pdf [file pmed.1003750.s012.pdf]

This paper describes a systematic review with meta–analysis of studies of mental health and homelessness.

I was asked for a statistical report and I interpret that to include all aspects of the design and conduct of the study.

Points of detail

Page 4 The studies which were found are primarily from high income settings. Is this a function of the choice of data–bases? Various empirical studies have indicated that the main bibliographic databases have varied and incomplete coverage of material not in English (Pilkington et al., 2005), and have limited coverage of material from low and middle–income country journals (Kieling et al., 2009; Syed Sheriff et al., 2008). Some of the WHO databases have a wider coverage. I have recently been advising a doctoral student who studied homelessness in Ethiopia so it clearly exists in at least one country in sub–Saharan Africa. (Her work is not yet published.)

Page 6 By PI here do the authors mean prediction interval or is it a typo for CI? Page 7 seems to suggest the latter and Ioannidis et al. (2007, authors' ref 31) is about confidence intervals for I^2. However in the results section we are indeed being given prediction intervals for the prevalences (which is clearly a good thing as Riley et al. (2011) have pointed out). I think an extra phrase is needed somewhere to clarify.

References It might be better to standardise the format for those not in English and perhaps add the language for those where the title is given in English. I suppose if you cannot recognise the language of a reference as German you are unlikely to send off for it but it might save people some work.

Points of more substance

I am not convinced that doing variable selection is a good idea here. Selecting a subset of variables in a way driven by the data leads to a model which is unlikely to replicate (Babyak, 2004). Screening variables to enter the model has been criticised too (Sun et al., 1996). The authors mention in the methods removing variables which had high correlations but I see no mention of this in the results section. Usually I would suggest using a model with all the possible moderator variables but with between 14 and 35 primary

studies per condition there is a limit to how many variables it is sensible to include in total.

Would the paper lose much if the multivariable models were removed? If the authors wish to include them a prominent health warning about their exploratory nature would be essential.

Summary

Mostly minor points. I think I would have given more prominence to the prediction intervals.

Michael Dewey

References

M A Babyak. What you see may not be what you get: a brief, nontechnical introduction to overfitting in regression–type models. *Psychosomatic Medicine*, 66:411–421, 2004.

J Ioannidis, N Patsopoulos, and E Evangelou. Uncertainty in heterogeneity estimates in meta–analyses. *British Medical Journal*, 335:914–916, 2007.

C Kieling, H Herrman, V Patel, and J J Mari. Indexation of psychiatric journals from low– and middle–income countries: a survey and a case study. *World Psychiatry*, 8:40–44, 2009.

K Pilkington, A Boshnakova, M Clarke, and J Richardson. 'No language restrictions' in database searches: what does this really mean? *The Journal of Alternative and Complementary Medicine*, 11:205–207, 2005.

R D Riley, J P T Higgins, and J J Deeks. Interpretation of random effects meta–analyses. *British Medical Journal*, 342:964–967, 2011.

G-W Sun, T L Shook, and G L Kay. Inappropriate use of bivariable analysis to screen risk factors for use in multivariable analysis. *Journal of Clinical Epidemiology*, 49:907–916, 1996.

R J Syed Sheriff, C E Adams, P Tharyan, M Jayaram, L Duley, and the PRACTIHC Mental Health Group. Randomised trials relevant to mental health conducted in low and middle–income countries: a survey. *BMC Psychiatry*, 8(69), 2008.
