# Supplementary material for: The prevalence of mental disorders among homeless people in high-income countries: An updated systematic review and meta-regression analysis
Source: PLoS Med. 2021 Aug 23;18(8):e1003750. doi: 10.1371/journal.pmed.1003750 (PMC8423293; doi:10.1371/journal.pmed.1003750)
Supplement: Attachment — Submitted filename: Detailed Response to Reviewers.docx [file pmed.1003750.s013.docx]

**Detailed responses to editorial comments and reviewers**

**Requests from the editors:**

**E1.** Abstract: Please report your abstract according to PRISMA for abstracts, following the PLOS Medicine abstract structure (Background, Methods and Findings, Conclusions) <http://www.plosmedicine.org/article/info:doi/10.1371/journal.pmed.1001419> .

**Response:** We reworked the abstract and made sure to check all the items of the PRISMA for Abstracts checklist.

**E2.** Abstract Background: Provide the context of why the study is important. The final sentence should clearly state the study question.

**Response:** The reworked Background section of the abstract now provides additional context and states the study question.

Abstract, Lines 27-32: “Homelessness continues to be a pressing public health concern in many countries, and mental disorders in homeless persons contribute to their high rates of morbidity and mortality. Many primary studies have estimated prevalence rates for mental disorders in homeless individuals. We conducted a systematic review and meta-analysis of studies on the prevalence of any mental disorder, and major psychiatric diagnoses in clearly defined homeless populations in any high-income country.”

**E3.** Abstract: Methods and Findings: Please provide the dates of search, data sources, types of study designs included, eligibility criteria, and synthesis/appraisal methods.

**Response:** Revised Methods and Findings sections now elaborate on these aspects.

Abstract, Lines 35-42: “We systematically searched for observational studies that estimated prevalence rates of mental disorders in samples of homeless individuals, using Medline, Embase, PsycInfo and Google Scholar. We updated a previous systematic review and meta-analysis conducted in 2007, and searched until April 1st 2021. Studies were included if they sampled exclusively homeless persons, diagnosed mental disorders by standardized criteria using validated methods, provided point or up to 12-month prevalence rates, and were conducted in high-income countries. We identified 39 publications with a total of 8049 participants. Study quality was assessed.”

**E4.** Abstract: Methods and Findings: Please conclude this section with a sentence describing the main limitation(s) of the study.

**Response:** Done.

Abstract, Lines 48-50: “Limitations included lack of information on certain subpopulations (women, immigrants) and unmet healthcare needs.”

**E5.** Abstract: Please include the study registration number in the Abstract.

**Response:** Done and funding information included.

Abstract, Lines 60-61: “This systematic review and meta-analysis has been registered with PROSPERO (CRD42018085216). SF is funded by the Wellcome Trust (202836/Z/16/Z).”

**E6.** Author Summary: At this stage, we ask that you include a short, non-technical Author Summary of your research to make findings accessible to a wide audience that includes both scientists and non-scientists. The Author Summary should immediately follow the Abstract in your revised manuscript. This text is subject to editorial change and should be distinct from the scientific abstract. Please see our author guidelines for more information: <https://journals.plos.org/plosmedicine/s/revising-your-manuscript#loc-author-summary>

**Response:** Done (see Author Summary: Lines 63-88).

**E7.** Introduction: Please expand on the rationale for the study: address past research and explain the need for and potential importance of the study.

**Response:** We have expanded on these parts of the introduction. See our response to R4.2 as well as the following:

Introduction, Lines 108-122: “Information on the overall extent and pattern of mental disorders among homeless people are necessary to inform resource allocation and service development, and allow for consideration of evidence gaps with researchers, clinicians, and policymakers. The large number of primary studies, of varying quality and samples, means that systematic reviews are required to clarify and synthesize the evidence, underscore main findings, and consider implications. According to a recent umbrella review, there have been at least seven systematic reviews with quantitative data synthesis in the past two decades (20); however, most of them focused on individual diagnostic categories (21–24), specific age bands (24,25) or were limited to a single country (26). The last meta-analysis to our knowledge which provided a comprehensive account of the prevalence of major mental disorders in homeless adults in high-income countries ended its search strategy in 2007 (27), and since then, a considerable number of primary studies have been published (28,29). Thus, we conducted an updated systematic review and meta-analysis on the prevalence of mental disorders among homeless people in high-income countries, and added the new diagnostic categories of any mental disorder and bipolar disorder.”

**E8.** Methods: Search Strategy: Please update the search to the present time (the end date is noted as October 2019).

**Response:** We agree with the editor that the review should not appear dated – so we decided to re-run the search, updating it until April 2021 (Methods, Line 131-133: “We have updated an earlier review (27) which was based on a search for articles published up until December 2007, so we targeted new primary studies published between January 2008 and March 2021”). We did not identify any more recent studies, though one paper published in 2009 has been included during the rechecking process. We re-performed the analyses and updated the results accordingly. The inclusion of this one additional study has had very little effect on the results (changing main prevalence estimates by a decimal place). The other changes to the findings that we outline below are secondary to improved statistical methods based on the helpful reviewer comments that we outline below (R3.1. and R3.2). This new search end date will increase the relevance of the paper for longer, and the new methods mean that the findings are more robust. Two very useful additions based on the review process.

**E9.** Methods: Please describe how you evaluated risk of bias, including publication bias.

**Response:** We used a standardized tool to assess risk of bias (Hoy et al. Assessing risk of bias in prevalence studies. J Clin Epidemiol. 201) as described in the “Data Collection and Quality Assessment” section (Methods, Line 176-178: “Each included publication was rated on methodological quality by two sets of criteria specifically designed to assess prevalence studies: the Joanna Briggs Institute Critical Appraisal Tool for Prevalence Studies (34) and a Risk of Bias Tool (35).”).
We conducted subgroup analyses to investigate whether studies evaluated as carrying low risk of bias had differing results. We came to the view that a separate assessment for publication bias was not necessary, see our response to R.4.7.

**E10.** Results: Line 191: If 5 of the studies did not report a participation rate, please clarify how was it determined whether the participation rate was 50% or greater (as outlined in the exclusion criteria)?

**Response:** On reflection, this phrasing of this exclusion criterion was misleading. In fact, we only excluded studies which explicitly reported a participation rate below 50% as this would likely bias the findings. Studies that did not report participation rate were typically not able to due to complex recruitment methods, but there was no indication from these studies that a majority of participants declined. We have now rephrased the criteria for exclusion to make this clearer:

Methods, Line 156-158: “Surveys which reported a response rate of less than 50% or exclusively sampled from selected subpopulations (such as elderly homeless, homeless youth, homeless single parents) were excluded.”

**E11.** Results: Line 256: Please clarify if “preferences” should be “prevalence” here.

**Response:** Thank you - an error on our part. This Line was removed when the Bipolar Disorders segment was revised as a whole (see Results, Lines 327-340)

**E12.** References: Please check formatting of references. Please us the "Vancouver" style for reference formatting, and see our website for other reference guidelines <https://journals.plos.org/plosmedicine/s/submission-guidelines#loc-references>
In Ref 24, Jama should be JAMA, for example.

**Response:** Done.

**E13.** Figure 1: It may be helpful to indicate at least broadly indicate reasons for exclusion in the flow diagram at screening points.

**Response:** We agree that indicating the reasons for exclusion, especially at the level of screening where full texts are assessed, is helpful. At the same time, we have avoided overloading this graphic with detailed explanations, especially considering the number of studies failing to meet more than one eligibility criterion. Please see our response to R4.5 on how we have dealt with this.

**E14.** Figures 2-8. For each figure, please provide a descriptive legend describing what is shown in the figure (for example, including the data points and bars, dotted line).

**Response**: Figure legends are now added to the figure captions embedded in the manuscript.

**E15**. PRISMA Checklist: Thank you for including the checklist as a supporting information file. Please revise the checklist, using section and paragraph numbers to refer to locations within the text, as opposed to page numbers.

**Response:** Reworked references to locations in the text are now included in the PRISMA checklist.

**E16.** S3 Table: Please define all abbreviations in a legend for the Table.

**Response:** Done (now S4 Table).

**E17**. S4 and S5 Tables: It would be helpful to include more descriptive title/legends for these tables.

**Response**: Agreed (now S5 and S6 Tables).

**E18**. S1 Text: Can you please explain why the results for affective disorders are being reported separately from the main text?

**Response:** The affective disorders category pools multiple diagnoses, including major depression and bipolar disorder, which other subsections of the results already report on. Therefore, we think it is mostly redundant if included in the main manuscript. So, we moved it into appendices.

**Reviewer #1**

This paper describes a systematic review with meta–analysis of studies of mental health and homelessness. I was asked for a statistical report and I interpret that to include all aspects of the design and conduct of the study.

Points of detail

**R1.1.** Page 4: The studies which were found are primarily from high income settings. Is this a function of the choice of data–bases? Various empirical studies have indicated that the main bibliographic databases have varied and incomplete coverage of material not in English (Pilkington et al., 2005), and have limited coverage of material from low and middle–income country journals (Kieling et al., 2009; Syed Sheriff et al., 2008). Some of the WHO databases have a wider coverage. I have recently been advising a doctoral student who studied homelessness in Ethiopia so it clearly exists in at least one country in sub–Saharan Africa. (Her work is not yet published.)

**Response:** It is an important comment to note that data from low- and middle-income locations is available and also researchers should use specific search strategies to identify this evidence. However, including only studies from high-income countries in this systematic review was a deliberate choice of ours (see Methods: Lines 115-116). Although we agree with the reviewer that homeless populations in developing countries is very relevant, we limited our investigations to high-income settings to obtain a sample of more comparable results, given that the coverage of social and medical services will be different in and vary widely between low- and middle-income nations, and the definition of homelessness within the context of these countries is more challenging (see Tipple & Speak. "Who is homeless in developing countries?: Differentiating between inadequately housed and homeless people." International Development Planning Review 28.1 (2006): 57-85.). In addition, homeless peoples in some low- and middle-income countries (LMIC) may be primarily refugee populations with specific mental health needs. Nevertheless, the point is well taken and we have added a comment to the limitations of the paper, the following comment:

Discussion, Lines 502-505: “The present review focuses on high income countries due to sample and diagnostic heterogeneity if a wider range of countries was included. It is important to note, however, that homeless populations in low and middle income countries need investigation, and may have higher rates of traumatic-related symptoms (110,111))”

**R1.2.** Page 6: By PI here do the authors mean prediction interval or is it a typo for CI? Page 7 seems to suggest the latter and Ioannidis et al. (2007, authors’ ref 31) is about confidence intervals for I 2 . However in the results section we are indeed being given prediction intervals for the prevalences (which is clearly a good thing as Riley et al. (2011) have pointed out). I think an extra phrase is needed somewhere to clarify.

**Response:** A very helpful comment. We agree that there was a lack of clarity about this. We did calculate prediction intervals for all our main meta-analytical models and we have now ensured to indicate this more clearly in the revised “Statistical Analysis” section.

Methods, Lines 197-201: “Additionally, we calculated 95% prediction intervals for all meta-analytical models (42). Because the ‘metafor::predict.rma’ function unrealistically assumes that the model variance τ2 were a known value (43), we instead implemented a method proposed by Higgins and colleagues which accounts for τ2 to be an estimate with limited precision ((44), expression 12).”

**R1.3.** References: It might be better to standardise the format for those not in English and perhaps add the language for those where the title is given in English. I suppose if you cannot recognise the language of a reference as German you are unlikely to send off for it but it might save people some work.

**Response:** We agree that references with a standardized format and titles that are translated into English, if necessary, make our sources more transparent to international readers. We reworked our bibliography accordingly.

Points of more substance

**R1.4.** I am not convinced that doing variable selection is a good idea here. Selecting a subset of variables in a way driven by the data leads to a model which is unlikely to replicate (Babyak, 2004). Screening variables to enter the model has been criticised too (Sun et al., 1996). The authors mention in the methods removing variables which had high correlations but I see no mention of this in the results section. Usually I would suggest using a model with all the possible moderator variables but with between 14 and 35 studies per condition there is a limit to how many variables it is sensible to include in total. Would the paper lose much if the multivariable models were removed? If the authors wish to include them a prominent health warning about their exploratory nature would be essential.

Summary: Mostly minor points. I think I would have given more prominence to the prediction intervals.

**Response:** These are valid criticisms of our original model selection and we revised our analyses accordingly. We narrowed down the number of independent variables tested in meta-regression analyses to six, which seems to us more fitting for the relatively small number of studies per meta-analytical model. Additionally, we abandoned the iterative, p-value-based model selection in the original version in favour of an information-criterion-based, automated procedure with R-package ‘glmulti’. For analyses with particularly low numbers of included studies (k<20), we utilized an information criterion designed to be robust against overfitting in small samples.

This makes our models more robust and reliable. We now outline this in the Methods:

Methods, Lines 218-222: “Multivariable meta-regression models were also calculated. The respective independent variables were chosen through automated, information-criterion-based model selection with generalized linear models (38). For models with 20 or more included studies, the Akaike information criterion (AIC) was used, for ones with less than 20 included studies we utilized the corrected version for small sample sizes (AICC) to avoid over-fitting.”

**Reviewer #2**

This manuscripts introduced a well conducted meta analysis and literature review study that aims to investigate the prevalence of mental disorders among homeless population in selected countries. The study is well conducted in that it used creditable guidelines and went through a solid process to retrieve fitting articles. It then used well recognized statistical method in data analysis. The writing of the paper in each section is clear. Discussion has its focus. The whole manuscript in writing and presenting key components of the study is cohesive and well focused. I do not see any particular errors, or mis-representations in writing. So, as for the study and its writing, I think it is publishable. So I examined this manuscript from scientific methods side of views.  Below are my comments to authors and to editors. You may make revisions if you accept my comments or leave them alone since the comments are more from my personal views.

**R2.1.** While it is important to continuously build up a profile of the prevalence in literature, reporting prevalence alone or as a main content of introduction for this paper, as presented mostly in statistics terms, seems a bit thin. The whole findings of the study as presented are prevalence rates, however detailed and broken down by the type of mental disorders.

**Response:** We agree that there are several relevant outcomes other than prevalence rates regarding the issue of mental disorders in homeless people, e.g., unmet mental healthcare needs or the effectiveness of support programs targeting the homeless mentally ill. At the same time, it is our view that prevalence is a first step to clarify these other important outcomes, and there is a need to be clear and rigorous on this matter. In addition, in our review, to add depth to the overall findings, we present an analysis of explanations for the differences in prevalence across studies. This is informative for future research, and also to interpret the prevalence findings. At the same time, we note the reviewer’s overall point in the revised Discussion:

Discussion, Lines 505-507: “Prevalence of the mental disorders reported in the current review does not consider unmet healthcare needs or treatment provision, which are additional elements to consider in developing services.”

**R2.2.** Only three countries are included. I understand this is because of the articles selected through a screening process. So it is not authors' purpose to include them only. Is there a way to explain/estimate why other, economically advanced, countries are not included?

**Response:** We think that there is a misunderstanding here. Studies from any high-income country were eligible for this systematic review, and we ended up including studies from 11 different countries, as indicated in the “Description of Included Studies” section (Results: Lines 246-252) as well as S4 Table: Study Characteristics.

**R2.3.** Readers like me would want to see prevalence rates of each of the countries for a comparison. This will add some contextual information to the paper.

**Response:** We chose not to display comparisons of prevalence estimates by each country in our analyses, possibly by subgroup analysis, because with included studies from 11 different countries this would have rendered our results too complicated. We did however include variables for the three countries that most studies originated from, the USA, UK and Germany, in our meta-regression analyses (and explain the reason for the choice of these countries). Additionally, the discussion includes some references to the prevalence rates of the general populations of the respective regions.

**R2.4.** As a research who has been working with homeless population, I also like to see a bit comparisons about demographic characteristics across the types of mental disorders, and countries.

**Response:** We extracted data on independent variables describing characteristics of the study samples, such as gender distribution, mean age and mean lifetime homelessness of participants, and these are now clarified in the revised manuscript (see S7 Table). However, we decided not to include stratify prevalence based on these demographic variables, with the exception of the gender variable, in our meta-regression analyses or in possible subgroup analyses in order not to overfit our models and to limit the length and complexity of our results section. We are concerned that they would throw up chance findings – partly as the level of detail on demographics reported in the primary studies varies and is limited. We have added this to the limitations as a future research area:

Discussion, Lines 496-500: “We limited the number of demographic variables that we conducted heterogeneity analyses due to variations in their measurement and reporting detail. Future work, including individual participant meta-analysis, could standardise information on age, socio-economic background, and ethnicity, for example.”

**Reviewer #3**

This is - in principle - a very useful and important update of the earlier review on mental disorders in people living homeless. However, the paper has two major methodological flaws related to the meta-analysis and meta-regressions that do not allow to proceed with the publication.

**R3.1.** Firstly, the variable selection procedure via p-value is as outdated as it is statistically wrong. If the authors want to stick to the model selection (there are many pros and cons related to the selection issue), they should use proper software solutions that are available in R metafor.

**Response:** This is a very helpful comment, and we are grateful to this reviewer for pointing us in the direction of ‘metafor’, a high quality and widely used software package. On reflection, we agree that iterative, p-value-based model selection is an obsolete method. We followed both these reviewer recommendations, reworked our analyses in R in order to implement ‘metafor’ and utilized automated, information-criterion-based model selection through the package ‘glmulti’. The “statistical analysis” section of our methods indicates these changes (Methods: Lines 182-230).

**R3.2.** Secondly, and also related to software issues, the authors should use a procedure that accounts for the problem of using multiple effects from the same study/publication. Again, R metafor is able to deal with this problem. In such situations, multilevel models (effects within studies/publications) should be conducted.

In the light of these problems, I suggest a rejection and re-submission after dealing with the issues.

**Response:** We went back and rechecked all the data extraction on the basis of this comment, and made sure only one sample/effect size per study is now included in any meta-analytical model. This avoids the need for employing multilevel models. Additionally, this comment raises the wider useful methodological point about the implications of extracting prevalence estimates of multiple diagnoses from the same samples, so we added to our limitations section:

Discussion, Lines 492-496: “With most of the primary studies reporting prevalence rates of more than one of the investigated diagnostic categories, effects from the same sample were in many cases entered into multiple meta-analytical models. This may have led to measurement error and overestimation if diagnostic criteria overlap, but without diagnostic validity studies specific to homeless persons, this remains uncertain.”

**Reviewer #4**

This is a well-structured meta-analytic review with robust methodology and profound significance for mental health research and policymaking in homeless populations. The presentation of the findings and articulation of the evidence in the context of homelessness appeared to be excellent. However, there are a few concerns that should be addressed to improve the scholarly value accuracy and implications of this manuscript.

**R4.1.** First, the abstract should clearly mention the objective of the review before stepping into the methods. Also, the abstract should inform that it is an updated review; therefore, mentioning the previous review and specifying the timeline of the current would substantially improve the abstract.

**Response:** We agree that these additions make our abstract more informative. We implemented both these suggestions in the reworked abstract. See our responses to the Editors’ notes E2-E4.

**R4.2.** Second, the introduction/background does not provide a clear understanding of why a review on high-income countries AND homeless people is required in the first place. It is essential to describe the "need" for a review. The authors may expand the biopsychosocial challenges associated with mental health in homeless people and how they are recognized amongst one of the most marginalized population groups in developed countries. Such explanations, among many others, are likely to inform the readers why this review is important and why we must focus on the evidence available on this critical issue. Also, multiple previous reviews on homelessness and mental health [1-4], not only the one that was published in 2007, should be discussed in the background to offer a broader understanding of the evidence landscape.

**Response:** This comment was very helpful in guiding us to expand on sections describing the rationale for our review and putting it into a wider research context. Accordingly, we have included additional information in the introduction, especially regarding previous reviews and how our paper aims to be more comprehensive than previous reviews, adding meta-regression analyses, new diagnostic categories, and incorporating a significant new body of new primary studies.

Introduction, Lines 112-119: “According to a recent umbrella review, there have been at least seven systematic reviews with quantitative data synthesis in the past two decades (20), however, most of them focused on individual diagnostic categories (21–24), specific age bands (24,25) or were limited to a single country (26). The last meta-analysis to our knowledge which provided a comprehensive account of the prevalence of major mental disorders in homeless adults in high-income countries ended its search strategy in 2007 (27), and since then, a considerable number of primary studies have been published (28,29).”

**R4.3.** Third, the authors should specify which mental disorders were considered before screening the articles. A complete list of disorders should accompany the manuscript, perhaps as supplementary material. Also, reference#22 was cited in the methods section, which was originally published in 1985. The authors may explain why they did not use the most contemporary definitions and categories of mental disorders as stated in DSM-5 or ICD-10. An informed author may wish to understand what disorders/conditions were within and beyond the scope of the current review.

**Response:** We agree that it should be made clearer which specific diagnoses were investigated. The list of prespecified disorders to be investigated is now more clearly stated at the top of the “Search Strategy” section, and also referenced in the eligibility criteria.

Methods, Lines 127-130: “1) schizophrenia spectrum disorders, (2) major depressive disorder, (3) bipolar disorder, (4) alcohol use disorders, (5) drug use disorders, (6) personality disorders, and (7) any current mental disorder (also known as axis-1-disorders in the DSM multiaxial system (30))”

What is now citation #30 was included to reference the DSM multiaxial system specifically (see Methods, Lines 129-130: “any current mental disorder (also known as axis-1-disorders in the DSM multiaxial system (30))”); it was not meant to indicate that we relied on partially obsolete definitions of specific disorders. Inclusion criterion (2) indicates that diagnoses could be defined by any iteration of ICD or DSM. Our included publications were published between 1979 and 2018, so naturally, some of them based their diagnostics on now obsolete versions of these diagnostic manuals. Retrospectively imposing the most recent definitions on their diagnostic categories is of course not possible.

**R4.4.** Fourth, the authors should consider sensitivity analyses to examine the effects of individual studies (and their effect sizes) on the pooled estimates for each meta-analytic model. Also, subgroup analyses in this review appeared to be limited to the risk of bias, whereas multiple subgroups could be constructed using variables such as gender (male vs. female), age (young adult vs. older adult), types of homeless (street-living vs. those in shelter homes), comorbidity (healthy vs. those with known medical conditions), geographic locations (homeless in North America vs. in Europe) etc. A set of subgroup analyses would be extremely helpful to interpret the differences in estimates in different subgroups and adopt specific measures to improve their mental health outcomes. This is important because homeless people are not homogenous [5-7]; therefore, their psychosocial stressors are likely to vary, and this must be recognized while estimating the burden of their mental health problems.

**Response:** We agree that homeless people form a heterogeneous group and many different factors might have an impact on an individual’s mental well-being. Methodologically, there are two major ways to investigate potential sources of heterogeneity in meta-analyses: Subgroup analyses, as proposed above, and meta-regression analyses. For the sake of conciseness, we focused on one of these methods for the examination of sample characteristics. We chose meta-regression models, the results of which are presented for every diagnostic category, because they allow the inclusion continuous variables instead of just categorial ones (so there would be no need for somewhat arbitrary cut-offs for variables like proportion of female participants or mean age of participants). Furthermore, we decided to trim the number of demographic and study characteristics investigated as independent variables down to six, in order to avoid overfitting our models. We do not anticipate that subgroup analyses would lead to different findings and can test this if requested.

**R4.5.** Fifth, the authors should provide explanations/reasons for the excluded studies, at least for those that were excluded during the full-text evaluation. That should inform how many articles were excluded for each specific reason(s).

**Response:** This is a valuable suggestion. We decided to add another table in the supplements stating the precise reasons for every exclusion at the final stage of screening. This makes our decisions about inclusion and exclusion more transparent.

Results, Line 235: “(see S3 Table for reasons for exclusion).”

**R4.6.** Sixth, the discussion section of this manuscript could offer more insights on what the current evidence informs about future research priorities. One of the goals of systematic reviews and/or meta-analysis is to identify research gaps and offer guidance on critical areas that require further assessment. Moreover, the interpretation of the evidence should accompany how the practitioners and policymakers can use the evidence in practice. Prevalence estimates can inform psychosocial vulnerabilities in the population of interest and inform the need for action that may address the existing disease burden. Such avenues must be explored in the discussion of the manuscript.

**Response:** We agree about the need for further research gaps to be highlighted and have added a number to the revision in the limitation section (including the need to understand unmet healthcare needs, for research synthesis in LMIC and for more details in primary studies on certain socio-demographic findings). As for further implications, we considered this carefully. In our view, we think that this would be more appropriate in a commentary, if the paper is accepted, as further implications will vary by country, region and service, and not strictly the findings of our review. We have focused on the most relevant implications of our findings for practitioners and policymakers to be the need for more integrated approaches in social and mental health care that account for the frequent co-occurrence of homelessness and mental illness. The fifth paragraph of our “Discussion” section highlights this point (Discussion: Lines 467-481).

**R4.7.** Lastly, the limitations of the review must be revisited and strengthened considering the following issues: a) a limited number of databases searched (with marked overlaps between similar databases such as Medline and PubMed), and b) a lack of reporting the publication bias (can be ignored in the authors provide the funnel plots/Egger's test estimates for publication bias).
References: *(…)*

**Response:** This is a helpful comment. We agree that point a) is a relevant addition, so we entered it into the reworked version of our limitations section (see Discussion: Lines 488-512).

Regarding point b), it is our opinion that an analysis on publication bias is not required for this specific systematic review. Publication bias refers to the preferential publication of significant results supporting an alternative hypothesis compared to possible results with other implications. If a prevalence estimate is the main outcome measure of a primary study, there is usually not a null hypothesis or an alternate hypothesis. We do not think that there are plausible scenarios in which a certain measured prevalence rate would have been less likely to be published.
