# Supplementary material for: The prevalence of mental disorders among homeless people in high-income countries: An updated systematic review and meta-regression analysis
Source: PLoS Med. 2021 Aug 23;18(8):e1003750. doi: 10.1371/journal.pmed.1003750 (PMC8423293; doi:10.1371/journal.pmed.1003750)
Supplement: Attachment — Submitted filename: Detailed Response to Reviewers_2nd_rev.docx [file pmed.1003750.s014.docx]

**Detailed responses to editorial comments and reviewers**

**Requests from the editors:**

**1.** Title: Please capitalize the first word of the subtitle, we suggest: “The prevalence of mental disorders among homeless people in high-income countries: An updated systematic review and meta-regression analysis”

**Response:** Done

**2.** Abstract: Line 41-42: Please provide slightly more detail on how study quality was assessed.

**Response:**

Abstract: Line 41-42: Study quality was assessed using the JBI Critical Appraisal Tool for Prevalence Studies and a Risk of Bias Tool.

**3.** Abstract: Line 54-56: We suggest revising to “Our findings suggest the burden of psychiatric morbidity in homeless persons is substantial…”

**Response:** Done

**4.** Author summary: “Why was this study done” Line 68, and Discussion: Line 436 and 512, 514, 518: We suggest replacing “the homeless” with “homeless individuals” or similar.

**Response:** Every instance of “the homeless” in the previous version has been rephrased.

**5.** Discussion: Line 477: Please spell out “RCT” in the text, if this is the first mention of the abbreviation.

**Response:** Done

**6.** Page 23: Please remove the “Funding” section from the main text and ensure all information is accurately entered in the “Financial Disclosure” section of the manuscript submission form.

**Response:** Done

**7.** References: Please double check that all references use the "Vancouver" style for reference formatting, and see our website for other reference guidelines https://journals.plos.org/plosmedicine/s/submission-guidelines#loc-references

Ref 22, 61, 66, 84, 96: Please double check if the reference information is complete. Please check the journal title abbreviation for Reference 102.

**Response:** All references have been checked and corrected. We completed the information regarding the above-mentioned references. All articles published in the Journal of the American Medical Association are now referenced with the correct abbreviation “J Amer Med Assoc”. References 61 and 66 have both been replaced by articles of the identical respective authors which were published in a better accessible, peer reviewed journal reporting the same data.

61. Kershaw A, Singleton N, Meltzer H. Survey of the health and well-being of homeless people in Glasgow. Int Rev Psychiatry. 2003;15(1–2):141–143.

66. Gill B, Meltzer H, Hinds K. The prevalence of psychiatric morbidity among homeless adults. Int Rev Psychiatry. 2003;15(1–2):134–140.

**8.** Figure 2 - 8: If possible, please increase the font size if possible, particularly along the X axis labels.

**Response:** The font sizes of the forest plots have been optimize with special attention to the readability of the X axis labels.

**9.** Supporting information files: Please include a “clean” version of each table and S1 Text.

**Response:** Done

**Comments from Reviewers:**

**Reviewer #1**: The authors have addressed my points successfully.

Michael Dewey

**Response**: We are grateful to this reviewer. No changes required.
